# Fosl1 is vital to heart regeneration upon apex resection in adult *Xenopus tropicalis*

**DOI:** 10.1038/s41536-021-00146-y

**Published:** 2021-06-29

**Authors:** Hai-Yan Wu, Yi-Min Zhou, Zhu-Qin Liao, Jia-Wen Zhong, You-Bin Liu, Hui Zhao, Chi-Qian Liang, Rui-Jin Huang, Kyu-Sang Park, Shan-Shan Feng, Li Zheng, Dong-Qing Cai, Xu-Feng Qi

**Affiliations:** 1 Key Laboratory of Regenerative Medicine of Ministry of Education, Department of Developmental & Regenerative Biology, Jinan University, Guangzhou, China; 2 Department of Cardiology, The Guangzhou Eighth People’s Hospital, Guangzhou, China; 3 School of Biomedical Sciences, Faculty of Medicine, The Chinese University of Hong Kong, Hong Kong, SAR China; 4 Department of Neuroanatomy, Institute of Anatomy, University of Bonn, Bonn, Germany; 5 Department of Physiology, Wonju College of Medicine, Yonsei University, Wonju, Gangwon Korea; 6 School of Environmental Science and Engineering, Guangdong University of Technology, Guangzhou, China

**Keywords:** Cell division, Experimental models of disease

## Abstract

Cardiovascular disease is the leading cause of death in the world due to losing regenerative capacity in the adult heart. Frogs possess remarkable capacities to regenerate multiple organs, including spinal cord, tail, and limb, but the response to heart injury and the underlying molecular mechanism remains largely unclear. Here we demonstrated that cardiomyocyte proliferation greatly contributes to heart regeneration in adult *X. tropicalis* upon apex resection. Using RNA-seq and qPCR, we found that the expression of Fos-like antigen 1 (Fosl1) was dramatically upregulated in early stage of heart injury. To study Fosl1 function in heart regeneration, its expression was modulated in vitro and in vivo. Overexpression of *X. tropicalis* Fosl1 significantly promoted the proliferation of cardiomyocyte cell line H9c2. Consistently, endogenous Fosl1 knockdown suppressed the proliferation of H9c2 cells and primary cardiomyocytes isolated from neonatal mice. Taking use of a cardiomyocyte-specific dominant-negative approach, we show that blocking Fosl1 function leads to defects in cardiomyocyte proliferation during *X. tropicalis* heart regeneration. We further show that knockdown of Fosl1 can suppress the capacity of heart regeneration in neonatal mice, but overexpression of Fosl1 can improve the cardiac function in adult mouse upon myocardium infarction. Co-immunoprecipitation, luciferase reporter, and ChIP analysis reveal that Fosl1 interacts with JunB and promotes the expression of Cyclin-T1 (Ccnt1) during heart regeneration. In conclusion, we demonstrated that Fosl1 plays an essential role in cardiomyocyte proliferation and heart regeneration in vertebrates, at least in part, through interaction with JunB, thereby promoting expression of cell cycle regulators including Ccnt1.

## Introduction

Heart failure is a leading cause of morbidity and mortality worldwide, due to a limited ability to regenerate the injured adult heart after myocardial infarction (MI)^1–4^. Although various strategies including cell-based and cell-free therapies are being explored to promote heart regeneration in animal models or human patients^5–8^, the efficacy of cardiac therapy and clinical implications remain uncertain^9^. It has been demonstrated that completely cardiac regeneration occurs in neonatal mouse heart after ventricular resection at 1 day after birth^10,11^. However, this regenerative capacity is lost at 7 day after birth, suggesting that regenerative potential is gradually lost during mouse heart development and maturation^10,11^. In contrast, lower vertebrates such as zebrafish and urodele amphibians retain a remarkable capacity to regenerate the injured heart in adulthood. The adult zebrafish can completely regenerate the injured heart after ventricular resection (~20%) primarily through cardiomyocyte (CM) proliferation^12–15^. Moreover, the adult urodele amphibians such as newt and salamander also retain a great competency to complete cardiac regeneration^16,17^. It has been demonstrated that mammalian CMs are able to divide and renew in adulthood in spite of the very lower frequencies^2,18,19^. Therefore, it is promising for mammalian heart regeneration to take advantage of mechanisms underlying heart regeneration of lower vertebrates. However, there is a distant evolutionary distance between the above lower vertebrates and mammalians. This defect may result in failure of mammalian heart regeneration when using mechanisms underlying heart regeneration of zebrafish, newt, and salamander.

Anuran amphibian frogs, including *Xenopus laevis* (African clawed frog) and *Xenopus tropicalis* (Western clawed frog), have closer evolutionary distance with mammalians compared with zebrafish and urodela, including newt and salamander. Both frogs have been demonstrated to completely regenerate spinal cord, tail, eye, and limb in tadpoles and/or froglets^20–23^. Tadpoles of *X. laevis* is able to regenerate the injured heart, but the heart regenerative capacity is reduced during metamorphosis and permanently lost even in 6-month-old juvenile frogs^24,25^. Although we previously revealed that adult *X. tropicalis* at age 1 year can regenerate the injured heart at 30 days post-resection (dpr)^26^, the underlying molecular mechanism is largely unclear. Given that *X. tropicalis* grows to adult in 4 months^27^, the 6-month-old *X. tropicalis* is really in adult stage. Importantly, 6-month-old *X. tropicalis* will save a half year time compared with the 1-year-old animal used in our previous study^26^. These disadvantages prompt us to ask whether and how 6-month-old *X. tropicalis* regenerates the heart following injury.

Activating protein-1 (AP-1) is a dimeric transcription factor typically comprised of the Fos and Jun family members. AP-1 has been reported to play important roles in many biological processes, including cell survival, apoptosis, cell differentiation, and cell proliferation^28,29^. Previous studies reported that AP-1 was significantly activated in congestive heart failure as well as in ischemic MI^30,31^. Furthermore, CMs bordering the infarct zone switch from a MEF2-driven homeostatic lineage specific to an AP-1-driven injury-induced gene expression program, which is required to prevent acute heart failure after infarction^32^. These reports revealed that AP-1 transcription factors have been implicated in cardiac function and cardiovascular diseases. A very recent study has shown that AP-1 transcription factor function in CMs is necessary for zebrafish heart regeneration by regulating chromatin accessibility changes, thereby promoting CM dedifferentiation, proliferation, and protrusion into the injured area^33^. Moreover, overexpression of Fosl1 and JunB leads to changes in behavior of CMs isolated from neonatal rat^33^. This recent study suggests that AP-1 transcription factors are critical for zebrafish heart regeneration. However, the functional importance of AP-1 members during *X. tropicalis* heart regeneration remains unclear.

To confirm this issue and further explore the underlying mechanism of *X. tropicalis* heart regeneration, we analyzed the gene expression profiles using RNA-sequencing (RNA-seq) and identified the potential regulators using bioinformatic analysis. We demonstrated that adult *X. tropicalis* is capable of regenerating heart without scarring after ventricular apex resection, mainly through proliferation of pre-existing CMs at least in part through the activation of Fosl1 pathway. The in vivo functional importance of Fosl1 during heart regeneration was also evidenced in *X. tropicalis* and mouse models. Our results propose that *X. tropicalis* is an alternative and suitable vertebrate model to study adult heart regeneration and suggest that Fosl1 is critical for the regulation of vertebrate heart regeneration.

## Results

### Regeneration of ventricular myocardium of *X. tropicalis* in adulthood

**Fig. 1:**
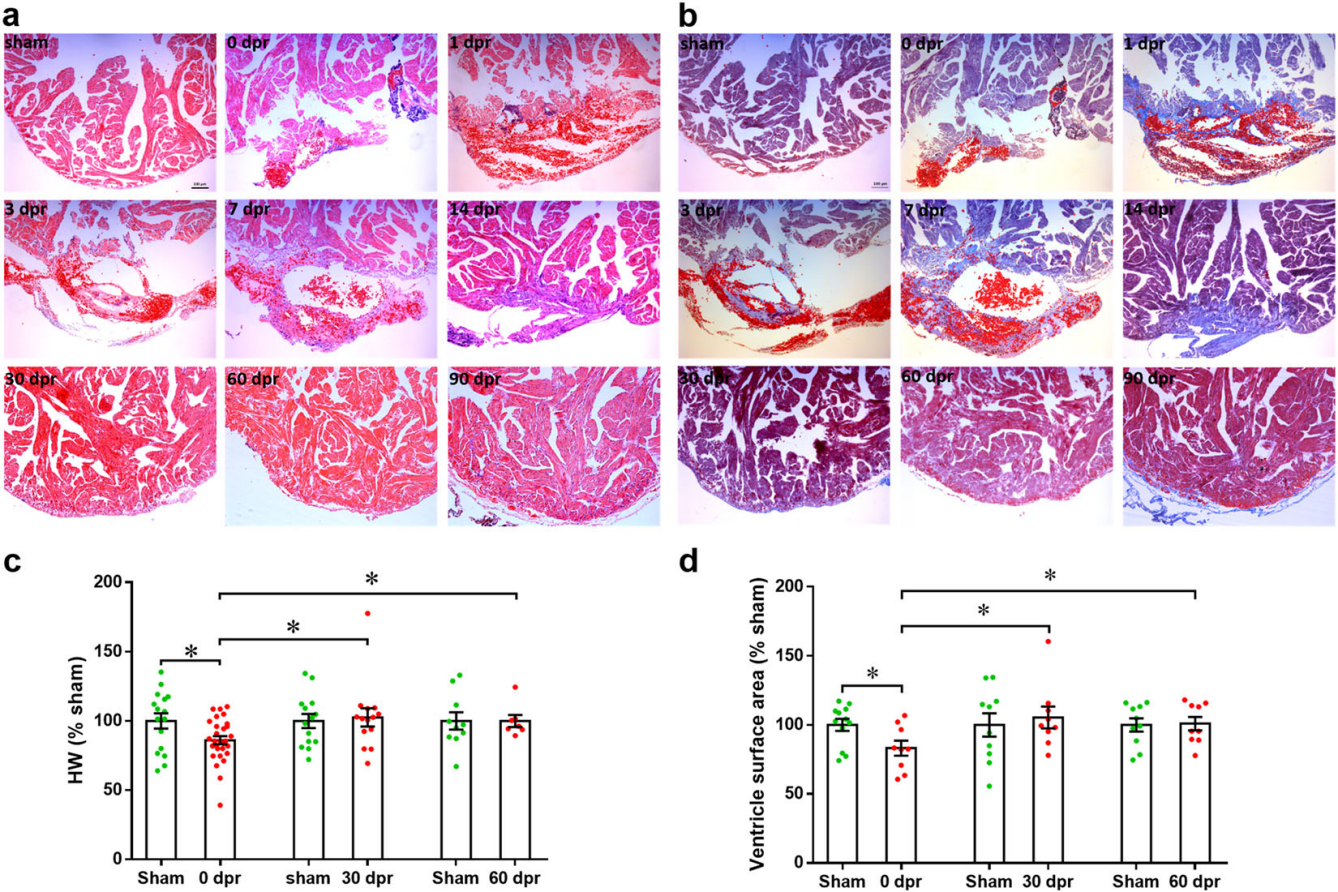
Regeneration of ventricular myocardium of *X. tropicalis*. **a** Hematoxylin and eosin (H&E) staining of the frog heart before resection (sham) or at 0, 1, 3, 7, 14, 30, 60, and 90 dpr. **b** Masson trichrome-stained sections showing early deposition of epicardial extracellular matrix at 1–14 dpr, with minimal evidence of cardiac fibrosis by day 30. **c**, **d** Quantification of regeneration at 0, 30, and 60 dpr. Ventricle weights (**c**) and sagittal section surface area (**d**) are presented as percentages of sham-operated controls. Numbers of samples analyzed are indicated within the bars. Data are presented as mean ± SEM, *p < 0.05 (Student’s *t* test).

A recent study has demonstrated that 6-month-old juvenile *X. laevis* permanently lose cardiac regenerative capacity^25^, prompting us to ask whether 6-month-old *X. tropicalis* can regenerate the heart following injury. Comparing these two species, *X. tropicalis* has a much shorter life cycle than does *X. laevis*, growing to adult in 4 months compared with 12 months^27^. Thus, 6-month-old *X. tropicalis* is really in adult stage. Serial histological analysis revealed progressive regeneration of the injured apex, with full restoration of the resected myocardium as early as 30 dpr (Fig. 1). Beginning at 1–7 dpr, the wound was sealed by a large blood clot in the apex (Fig. 1a, b), which was associated with a robust inflammatory response characterized by erythrocytes and mononuclear cells (Fig. 1a, b and Supplementary Fig. 1), in the similar manners of the 1-year-old *X. tropicalis* as previously reported^26^. Subsequent time points shown gradual re-sorption of the apical blood clot and its replacement by normal myocardial tissue. By 30 dpr, the entire apical defect was replaced by CMs, as detected by morphological analysis (Fig. 1a, b). From 1–14 dpr, infilled erythrocytes and inflammatory cells were replaced by fibrin, which reached maximum levels at 14 dpr and completely disappeared by 30 dpr (Fig. 1b and Supplementary Fig. 1b). Histological analysis showed that the size and shape of ventricles appeared grossly normal by 30 dpr (Fig. 1 and Supplementary Figs. 2 and 3).

To quantify the regeneration of *X. tropicalis* heart, we analyzed heart weight (HW) and surface area with or without resection. About 14% of HW or 17% of ventricular section surface area was removed and both were completely recovered by 30 dpr (Fig. 1c, d and Supplementary Fig. 2a–f). Moreover, there was no significant difference in HW/body weight (BW) ratio between the resected and sham-operated groups at 30 and 60 dpr (Supplementary Fig. 2d). It should be noted that both BW and HW have no significant difference between the resected and sham groups prior to ventricle resection at 0 dpr (Supplementary Fig. 2a–c). Given that individual frogs showed incomplete regeneration (IR) at 30 dpr (Supplementary Fig. 2f, right), a statistical approach was used to evaluate the overall proportion of complete regeneration (CR), which were characterized by the intact apex restoration. Results showed that 73.3, 76.5, and 100% hearts with resection were completely regenerated at 30, 60, and 90 dpr, respectively (Supplementary Fig. 2g). These data suggest that repairing period plays important roles in heart regeneration of *X. tropicalis*. One of the key characteristics of myocardial regeneration is the restoration of normal contraction. Therefore, we assessed the systolic properties of beating heart by visual inspection at 7–30 dpr and further confirmed the normal systolic function of regenerated apex at 30 dpr (Supplementary Fig. 3 and Supplementary Videos 1–4).

### CM proliferation contributes to heart regeneration of *X. tropicalis*

**Fig. 2:**
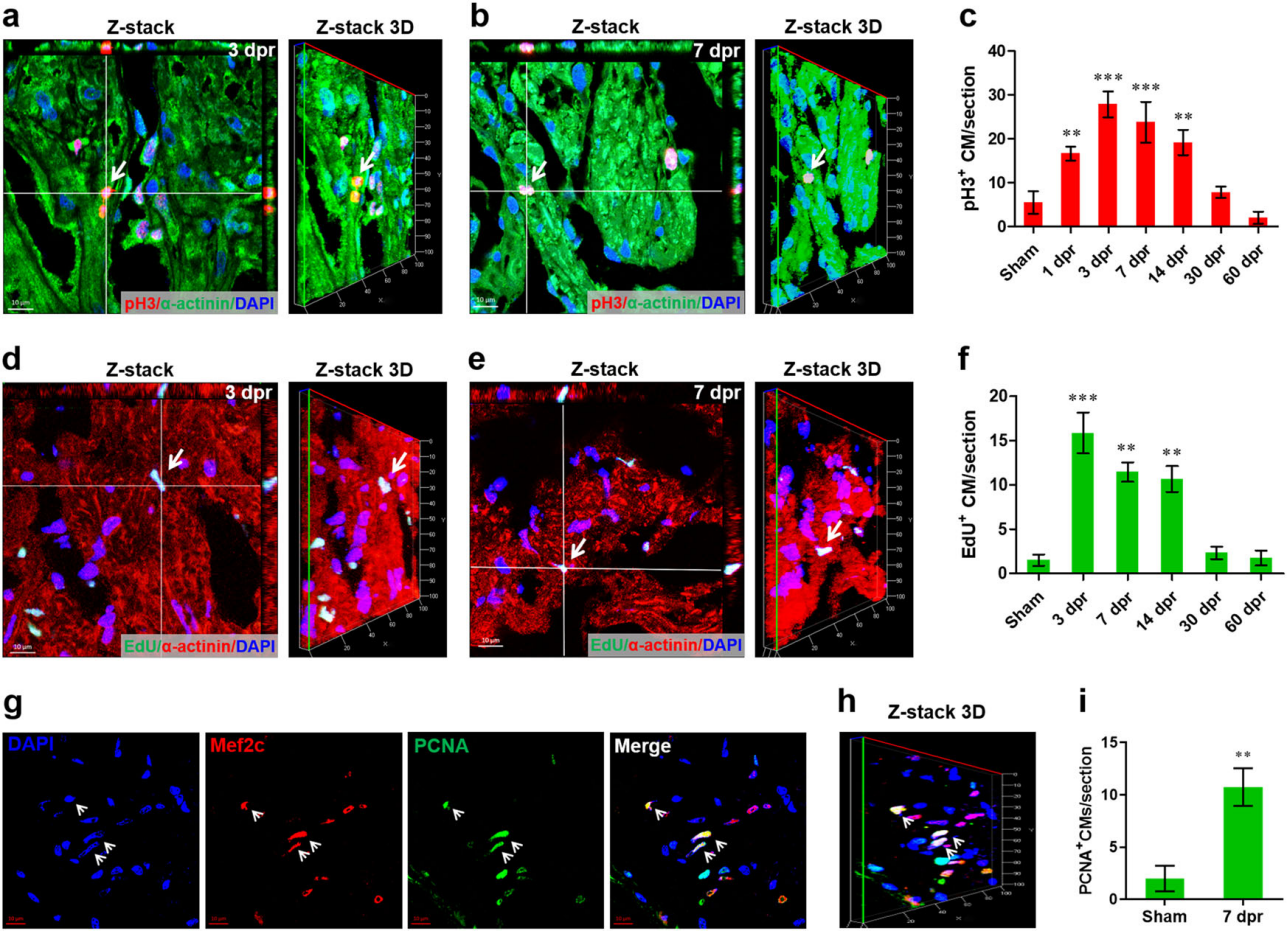
Surgical injury induces *X. tropicalis* heart regeneration through cardiomyocyte proliferation. **a**, **b** Representative *Z*-stack confocal images of pH3^+^ cardiomyocytes (arrow) at 3 (**a**) and 7 (**b**) dpr. **c** Quantification of pH3^+^ cardiomyocytes during heart regeneration within 60 days. Data are presented as mean ± SEM (*n* = 4 each), **p < 0.01, ***p < 0.001 versus sham (one-way ANOVA plus Dunnett’s test). **d**, **e** Representative *Z*-stack confocal images of EdU^+^ cardiomyocytes (arrow^)^ at 3 (d) and 7 (**e**) dpr. **f** Quantification of EdU^+^ cardiomyocytes during heart regeneration within 60 days. Data are presented as mean ± SEM (*n* = 4 each), **p < 0.01, ***p < 0.001 versus sham (one-way ANOVA plus Dunnett’s test). **g**–**i** Representative confocal (**g**) and *Z*-stack (**h**) images of PCNA^+^ cardiomyocytes (arrow) at 7 dpr with the quantification (**i**). Data are presented as mean ± SEM (*n* = 4 each), **p < 0.01 versus sham (Student’s *t* test).

Given that the restoration of cardiac muscle may result from myocyte hypertrophy or proliferation, we first assessed the size of newly formed CMs in apex and revealed no difference between the resected and sham groups at 30–60 dpr (Supplementary Fig. 4a–d). Moreover, there were no differences in the expression of hypertrophic genes^10,34,35^ in the hearts at 30 and 60 dpr compared with sham-operated hearts (Supplementary Fig. 4e). Therefore, we further analyzed the potential proliferative capacity of CMs in apex using colocalization of phosphor-histone H3 (pH3) with α-actinin. As shown in Fig. 2a, b, typical proliferating response of CMs in the apex at 3 and 7 dpr was confirmed by *Z*-stack imaging of pH3^+^ α-actinin^+^ cells. Moreover, time course of apex resection showed a significant increase in the number of pH3^+^ CMs in apex at 1–14 dpr, and the maximum level was observed at 3 dpr (Fig. 2c and Supplementary Fig. 5a). Subsequently, we assessed the cell cycle entry of CMs by measuring the nuclear incorporation of 5-ethynyl-2’-deoxyuridine (EdU), an efficient marker of DNA synthesis (Supplementary Fig. 5b). As expected, the representative EdU incorporation in CMs at 3 and 7 dpr was captured by confocal microscopy and confirmed by *Z*-stack confocal images (Fig. 2d, e). In control hearts, almost no EdU^+^ α-actinin^+^ cells (EdU^+^ CMs) were detected. However, significant increases in the number of EdU^+^ CMs were detected near the resection plane at 3–14 dpr and, the EdU incorporation peaked at 3 dpr. By 30 dpr, the EdU incorporation index in apex considerably decreased to the level as in sham-operated hearts (Fig. 2f and Supplementary Fig. 5c). In addition, proliferating CMs at 7 dpr were also confirmed by proliferating cell nuclear antigen (PCNA) and myocyte enhancer factor 2C (Mef2C) double staining (Fig. 2g–i). For cells absent from G0 phase, EdU specifically labels DNA synthesis in cell cycle stage of S, pH3 is specifically expressed in G2 and M stages, and PCNA is specifically expressed in all the cell stages of G1, S, G2, and M^36^. Thus, injury-induced proliferation of CMs in adult *X. tropicalis* was systematically evidenced in the present study.

**Fig. 3:**
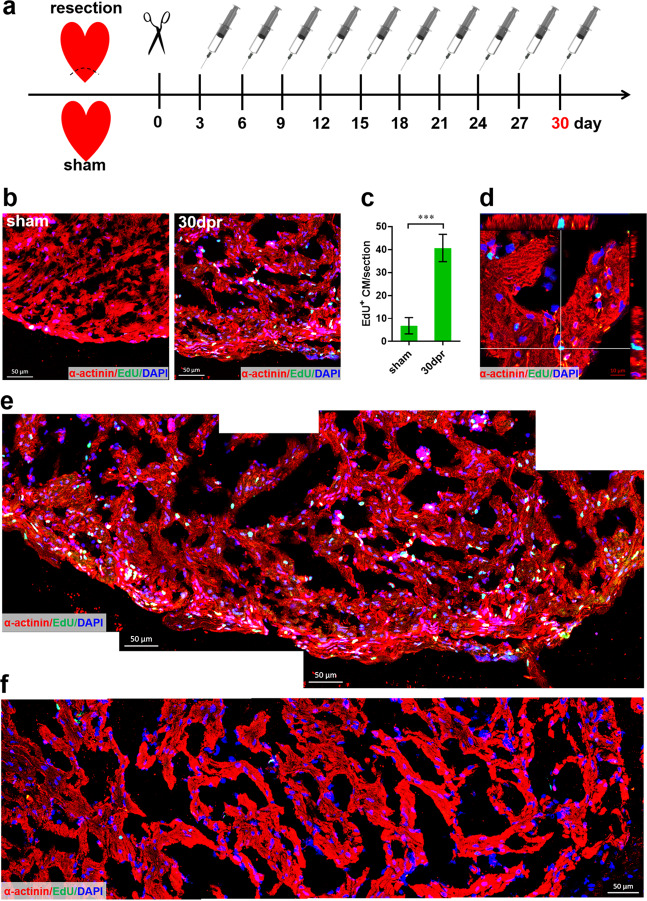
Proliferation of cardiomyocytes contribute to the regenerated ventricle apex in *X. tropicalis*. **a** Schematic of EdU pulse-chase experiment designed to label proliferating CMs during regeneration. **b**–**d** Representative images (**b**) and quantification (**c**) of EdU^+^ cardiomyocytes in the ventricle apex following EdU injection at 30 dpr revealing a great contribution of the pre-existing cardiomyocytes proliferation to apex regeneration. Green indicates EdU; red, α-actinin; blue, nuclei. Data are presented as mean ± SEM (*n* = 4 for sham and 8 for 30 dpr pulse), ***p < 0.001 (Student’s *t* test). Representative *Z*-stack confocal images of EdU^+^ cardiomyocytes are shown (**d**). **e**, **f** Combination of representative confocal images at high magnification showing substantial EdU^+^ cardiomyocytes in the regenerated ventricle apex (**e**) but not in the remote zone (**f**).

To further determine whether the regenerated apex contains newly formed CMs, a pulse-chase experiment was used after apex resection (Fig. 3a). After the pulse, greatly increased EdU^+^ CMs were observed in the regenerated apex at 30 dpr but not in the remote zone (Fig. 3b–f and Supplementary Fig. 6), which revealed that the regenerated myocardium results, at least in part, from the proliferating and newly forming CMs. These findings in *X. tropicalis* partially support the idea that the activation and/or proliferation of pre-existing CM subpopulations contributes to the heart regeneration in adult zebrafish^13,14^.

### Variations in injury-regulated genes in the *X. tropicalis* heart

**Fig. 4:**
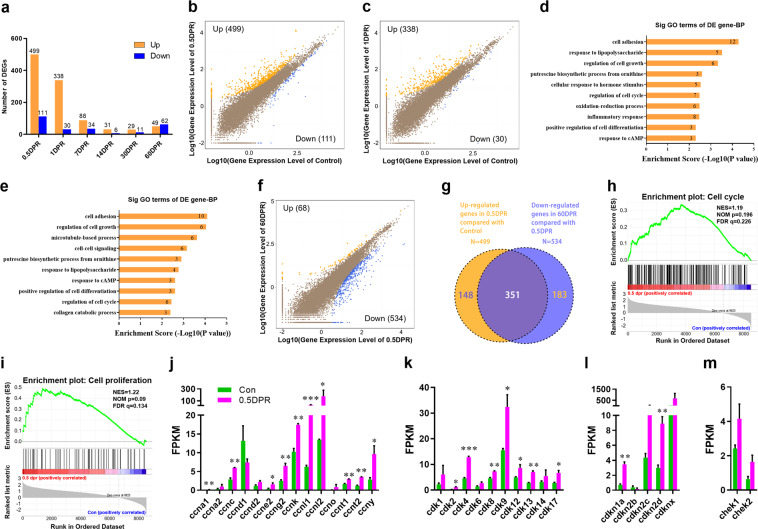
RNA-seq analysis for sham-operated and resected ventricles of *X. tropicalis*. **a** Statistic of differentially expressed genes in resected ventricles compared to control. **b**, **c** Overall changes of genes in resected ventricles at 0.5 dpr (**b**) and 1 dpr (**c**) compared to controls. **d**, **e** GO analysis of differentially expressed genes in ventricles at 0.5 dpr (**d**, 499 plus 111 genes) and 1 dpr (**e**, 338 plus 30 genes) over controls. **f** Overall change of genes in resected ventricles at 60 dpr compared to 0.5 dpr. **g** Upregulated genes in 0.5 dpr compared with control (499) overlaid with downregulated genes in 60 dpr compared with 0.5 dpr (534). **h**, **i** GSEA analysis of heart with apical resection at 0.5 dpr showing high correlations with cell cycle (**h**) and proliferation (**i**) gene sets. **j**–**m** Expression of selected genes relative to cyclin (**j**), cyclin-dependent kinase (**k**), cyclin-dependent kinase inhibitor (**l**), and checkpoint kinase (**m**) was analyzed by using the FPKM levels from RNA-seq. All results are presented as mean ± SEM (*n* = 3), *p < 0.05, **p < 0.01, ***p < 0.001 versus control (Student’s *t* test).

To identify target genes involved in heart regeneration in *X. tropicalis*, we collected the resected ventricles from 0.5 to 60 dpr and performed RNA-seq analysis. Most differently expressed genes were detected in the early stage (0.5 and 1 dpr) of heart injury (Fig. 4a). We identified 610 (499 up and 111 down) and 368 (338 up and 30 down) differently expressed genes at 0.5 and 1 dpr, respectively (Fig. 4b, c). Gene ontology (GO) analysis revealed that these differently expressed genes at 0.5 and 1 dpr were significantly enriched in gene sets involved in cell growth and cell cycle regulation (Fig. 4d, e), suggesting the involvement of cell proliferation in response to apex resection. Given that *X. tropicalis* completes the heart regeneration upon 30 dpr (Fig. 1 and Supplementary Fig. 3), potential targets responsible for heart regeneration (especially in early stage) will return to normal levels after 30 dpr. As expected, there was extensive overlap (351) between the upregulated genes at 0.5 dpr (versus sham) and the downregulated genes at 60 dpr (versus 0.5 dpr) (Fig. 4b, f, g), implying that the differently expressed genes in the early stage of heart injury contains the potential targets responsible for heart regeneration. In consistent with this finding, the gene set enrichment analysis (GSEA) using the control and 0.5 dpr groups revealed that both cell cycle and proliferation are highly related to the response to heart injury in adult *X. tropicalis* (Fig. 4h, i). Indeed, RNA-seq analysis revealed significant increases in the expression levels of substantial cell cycle-related genes, including cyclin, cyclin-dependent kinase, and checkpoint kinase, at 0.5 dpr compared to control (Fig. 4j–m).

### Fosl1 interacts with JunB and promotes *ccnt1* expression in *X. tropicalis*

**Fig. 5:**
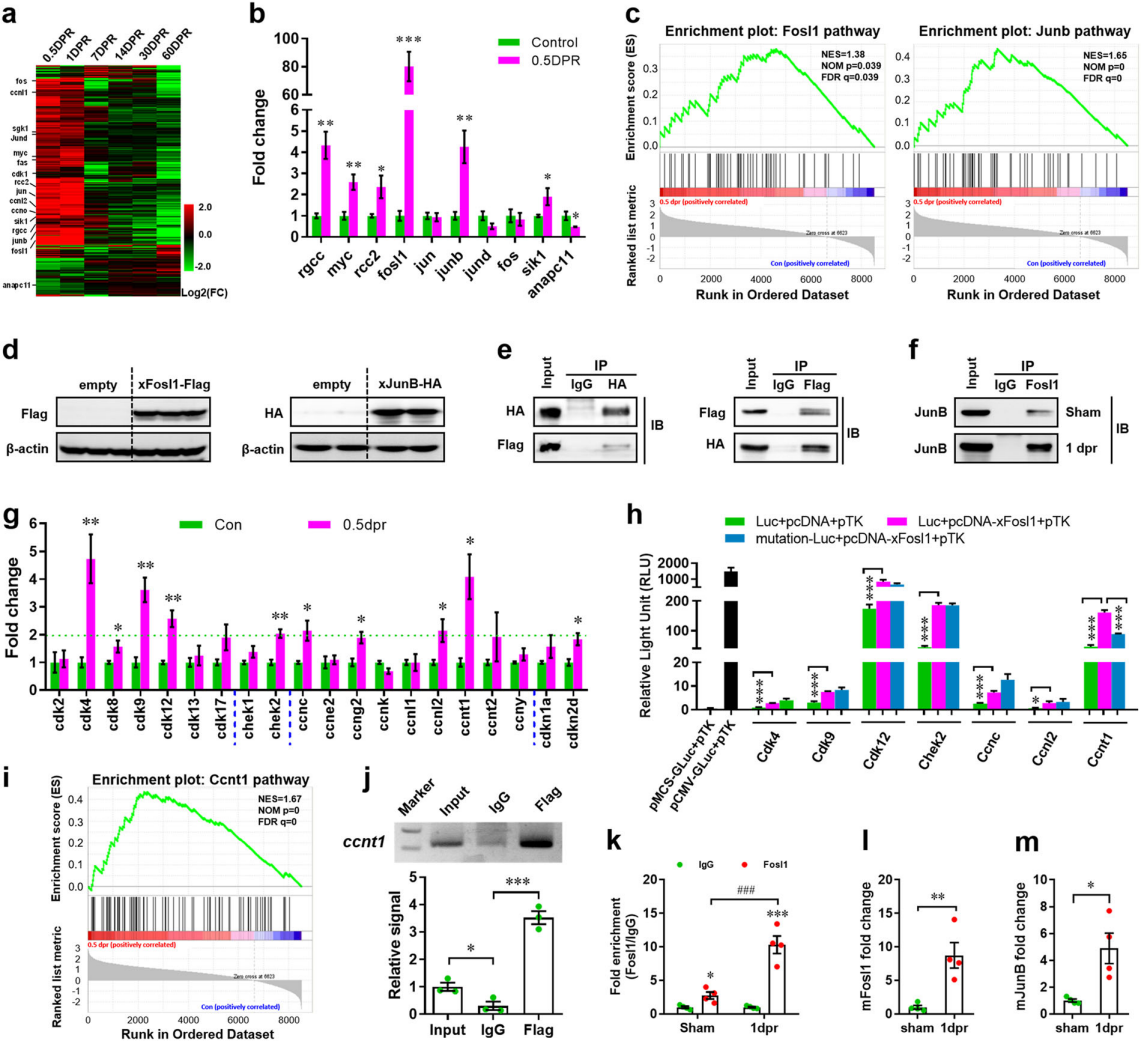
Fosl1 interacts with JunB and promotes *ccnt1* expression during heart regeneration in *X. tropicalis*. **a** Heat map of log2(FC) values showing genes differentially expressed in the hearts at 0.5, 1, 7, 14, 30, and 60 dpr compared with sham. **b** The qPCR of some selected regulators for cell cycle progression in sham and 0.5 dpr hearts (*n* = 4 each). **c** GSEA analysis of hearts at 0.5 dpr showing high correlations with Fosl1 (left) and JunB (right) pathways. **d** Expression of Flag-tagged xFosl1 (left) and HA-tagged xJunB (right) in *X. tropicalis* embryos was confirmed by western blotting. **e** CoIP assay showing the interaction between xFosl1-Flag and xJunB-HA in *X. tropicalis* embryos. **f** CoIP analysis showing the increased interaction between Fosl1 and JunB in regenerating neonatal mouse heart compared with the quiescent heart. **g** qPCR validation of cell cycle regulators with significant upregulation in RNA-seq analysis (*n* = 4 each). **h** Promoters of the cell cycle regulators with more than twofold upregulation were subjected to luciferase activity assay, using reporter plasmids and mutants (*n* = 5 each). **i** GSEA analysis of the hearts at 0.5 dpr showing high correlations with Ccnt1 pathway. **j** Representative image of ChIP assay confirmed the in vivo interaction of Fosl1 with promoters of *ccnt1* in *X. tropicalis* embryos. **k** ChIP-qPCR analysis showing the increased interaction between Fosl1 protein and Ccnt1 promoter region in regenerating neonatal mouse heart compared with quiescent heart. **l**, **m** qPCR validation of *Fosl1* (**l**) and *JunB* (**m**) in sham and resected hearts of neonatal mice (*n* = 4 each). All data are presented as mean ± SEM, *p < 0.05, **p < 0.01, ***p < 0.001 versus control (Student’s *t* test).

It has been demonstrated that proliferation of the pre-existed CM greatly contributes to the heart regeneration in both zebrafish and neonatal mice^10–14^. In our model, substantial proliferation-related genes were significantly increased at early stage of apex resection (Fig. 5a). The quantitative real-time PCR (qPCR) further confirmed that Fos-like antigen 1 (*fosl1*) expression was elevated to the highest level (>80-fold) by apex resection (Fig. 5b). In situ hybridization showed very faint signals for *Fosl1* probe in the heart apex from sham-operated *X. tropicalis*. However, great increase in *Fosl1* expression was detected in the apical ventricle at 0.5 dpr (Supplementary Fig. 7a). In consistent with the findings during heart regeneration in *X. tropicalis*, immunofluorescent staining revealed the increased expression of Fosl1 in CMs located in the heart apex from neonatal mouse at 0.5 dpr (Supplementary Fig. 7b, c). To define which cell types express Fosl1 during *X. tropicalis* heart regeneration, we further determined the expression pattern of Fosl1 in CMs and non-CMs (nCMs). During heart regeneration, a greatly different expression pattern of Fosl1was detected between CMs and nCMs isolated from the hearts at 0.5 dpr. Importantly, Fosl1 expression levels in CMs are significantly higher than that in nCMs during heart regeneration (Supplementary Fig. 7d). Therefore, these findings indicate that the great upregulation of Fosl1 during *X. tropicalis* heart regeneration dominantly results from CMs. This idea was further confirmed during neonatal mouse heart regeneration. In the non-injured heart at 0 dpr, Fosl1 expression levels in CMs are greatly higher than that in nCMs. There was no significant difference in Fosl1 expression levels between CMs and nCMs isolated from the injured heart at 0.5 dpr, although heart injury increased Fosl1 expression in both CMs and nCMs (Supplementary Fig. 7e). Given that CMs are the maximum cell type in whole heart and has the highest percentage in all heart cells^37^, it is reasonable to propose that CMs are likely to be the main cell type to express Fosl1 during neonatal mouse heart regeneration.

As a component of AP-1 complex, Fosl1 action requires a dimerization through interaction with JunB in mammals^38^. In our model, the increased expression of *fosl1* was accompanied by *junb* in the injured *X. tropicalis* hearts (Fig. 5a, b). Moreover, GSEA analysis indicated that both Fosl1 and JunB pathways are significantly related to the early heart injury and repair responses in adult *X. tropicalis* (Fig. 5c). Co-immunoprecipitation (CoIP) further confirmed that Flag-tagged *X. tropicalis* Fosl1 (xFosl1-Flag) indeed interacts with HA-tagged *X. tropicalis* JunB (xJunB-HA) in *X. tropicalis* embryos (Fig. 5d, e). The direct interaction between Fosl1 and JunB was further confirmed in the regenerating neonatal mouse heart (Fig. 5f). Taken together, these data imply that increased Fosl1 expression might contribute to CM proliferation during heart regeneration.

To further explore the potential target genes of Fosl1 during heart regeneration in *X. tropicalis*, significantly upregulated cell cycle-related genes in RNA-seq assay (Fig. 4j–m) were confirmed by qPCR analysis. We screened seven potential targets (*cdk4*, *cdk9*, *cdk12*, *chek2*, *ccnc*, *ccnl2*, and *ccnt1*) with more than twofold upregulation in the resected hearts for further analysis (Fig. 5g). The potential binding sites of FOSL1::JUNB transcription factor complex were predicted and identified in the promoter regions of target genes (Table S5). Luciferase reporter assay showed that overexpression of *xFosl1* significantly increased luciferase activities driven by promoters of all the seven target genes. Mutation of the reporter plasmids further confirmed that *ccnt1* is likely to be directly regulated by Fosl1 in *X. tropicalis* (Fig. 5h). GSEA analysis further confirmed that Ccnt1 (*p* = 0, false discovery rate = 0) pathway is significantly related to the heart injury and regeneration in *X. tropicalis* (Fig. 5i). The in vivo direct interaction between Fosl1 and the promoters of *ccnt1* was further confirmed by chromatin immunoprecipitation–polymerase chain reaction (ChIP-PCR) assay using *X. tropicalis* embryos (Fig. 5j). Due to the lacking of specific Fosl1 antibody for *X. tropicalis*, we performed an in vivo ChIP-qPCR assay using the regenerating heart from neonatal mouse to directly check the interaction of Fosl1 with promoters of Ccnt1 during heart regeneration. As shown in Fig. 5k, Fosl1 can significantly precipitate the promoter region of Ccnt1 in the uninjured neonatal mouse heart compared with immunoglobulin G (IgG). However, Fosl1-precipitated Ccnt1 promoter region was greatly increased in the injured heart compared with IgG control protein (Fig. 5k). These findings are consistent with the increased production of Fosl1 and JunB in the injured hearts (Fig. 5l, m). Taken together, these findings suggest that Fosl1 promotes CM proliferation by upregulating *ccnt1* expression through interaction with JunB, thereby contributing heart regeneration.

### Fosl1 promotes CM proliferation in mammals

**Fig. 6:**
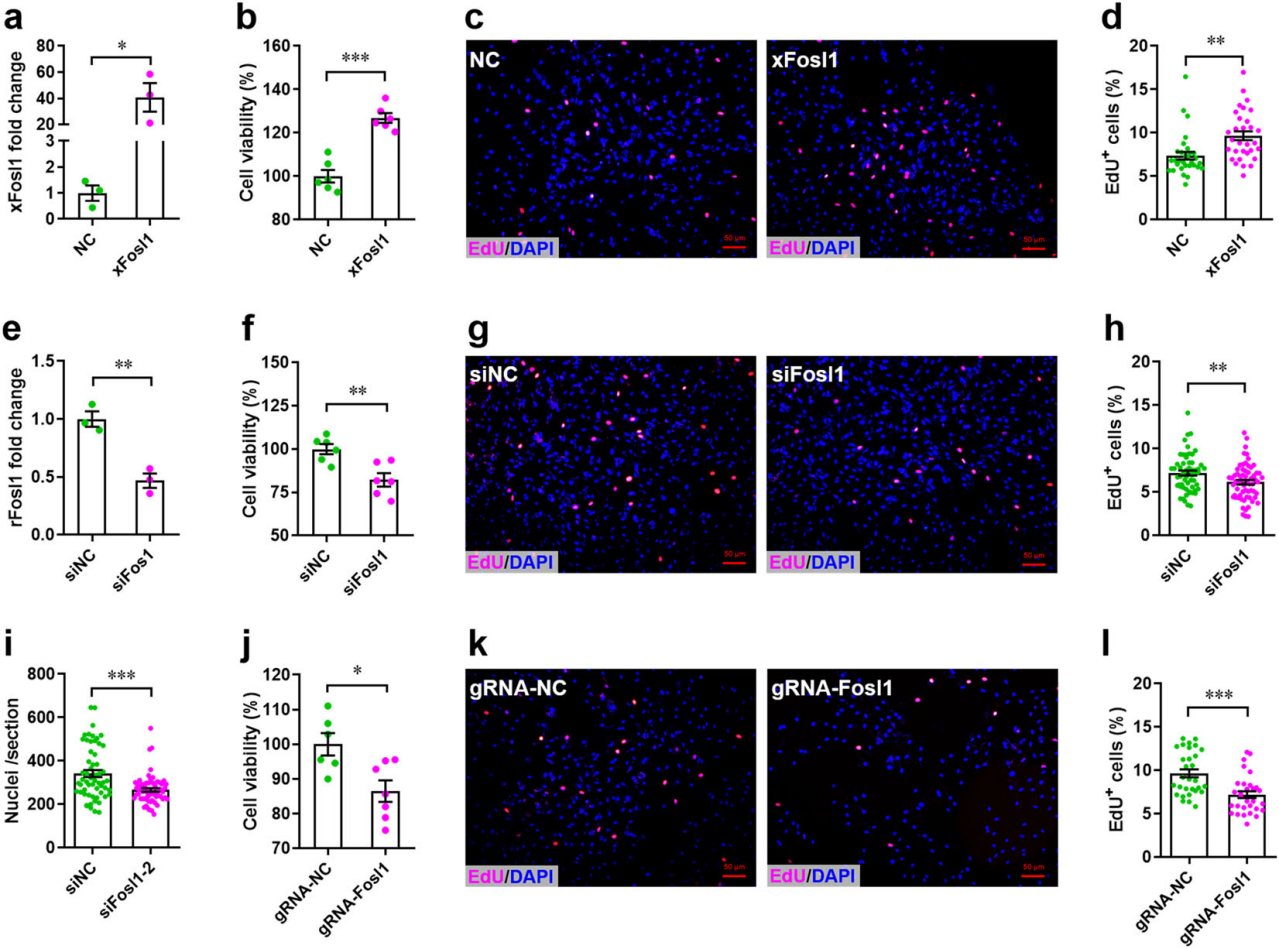
Fosl1 is critical to the proliferation of H9c2 cells. **a**–**d** H9c2 cells were transfected with pLOX-xFosl1 or pLOX-NC for 48 h, followed by qPCR validation of *X. tropicalis* Fosl1 (*xFosl1*) expression (**a**, *n* = 3 each), cell counting assay (**b**, *n* = 6 each), and nuclear EdU incorporation assay, respectively. Representative images (**c**) and quantification (**d**) of EdU^+^ cells are shown (*n* = ~30 fields from 5 wells per group). **e**–**i** Endogenous rat Fosl1 (rFosl1) expression in H9c2 cells was silenced using siRNA for 48 h, followed by qPCR validation (**e**), cell counting assay (**f**), and EdU incorporation assay. Representative images (**g**) and quantification (**h**) of EdU^+^ cells as well as quantification of total cells (**i**) are shown (*n* = ~60 fields from 10 wells per group). **j–l** Stable Fosl1 knockdown H9c2 cell line was established using LentiCRISPRv2 system. Cell counting assay (**j**, *n* = 6–7 each), representative images (**k**), and quantification (**l**) of EdU^+^ cells are shown (*n* = ~30 fields from 5 wells per group). All data are presented as mean ± SEM. *p < 0.05, **p < 0.01, ***p < 0.001 (Student’s *t* test).

Previous studies have demonstrated that transcription factor Fosl1 promotes proliferation of human lung adenocarcinoma cells^39^ and differentiation of mouse embryonic stem cells^40^. To determine whether Fosl1 influences proliferation of CMs, H9c2 cells (a rat CM cell line) were transfected with lentivirus overexpressing Fosl1, followed by cell count assay and EdU incorporation assay. We found that overexpression of Fosl1 (Fig. 6a) greatly promoted cell growth compared with negative control (Fig. 6b). In consistent with this, the percentage of EdU^+^ cells was elevated by overexpression of Fosl1 (Fig. 6c, d). These data suggest that Fosl1 promotes proliferation of H9c2 cells. Subsequently, we silenced the endogenous Fosl1 in H9c2 cells using small interfering RNA (siRNA; Fig. 6e) and found that Fosl1 silencing led to decrease in proliferation of H9c2 cells as demonstrated by cell count assay (Fig. 6f) and EdU incorporation assay (Fig. 6g–i). In addition, we further established a stable *Fosl1* knockdown H9c2 cell line using LentiCRISPRv2 system (Supplementary Fig. 8) and found that *Fosl1* deficiency indeed suppressed proliferation of H9c2 cells (Fig. 6j–l).

**Fig. 7:**
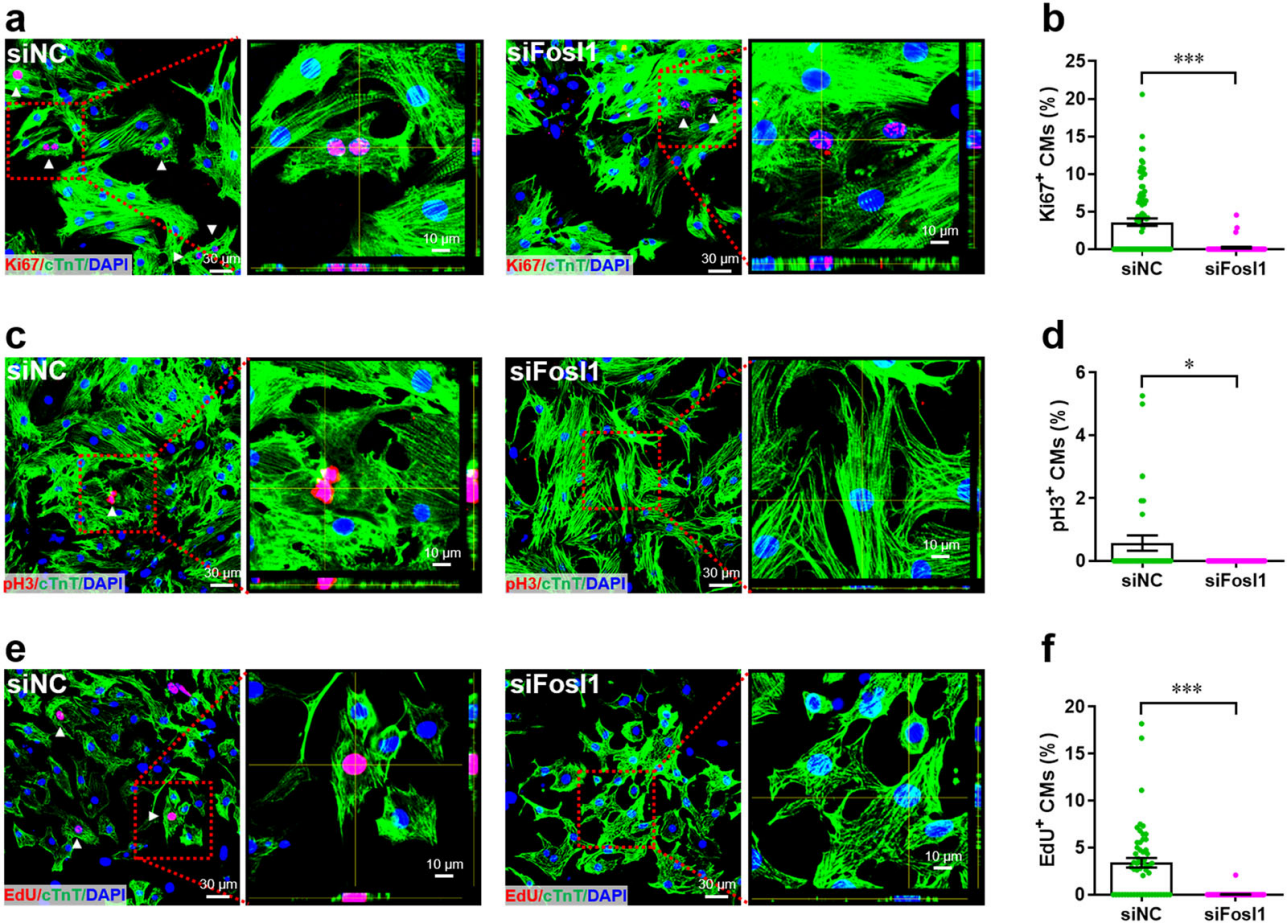
Fosl1 is required for the proliferation of primary cardiomyocytes. **a**, **b** Primary cardiomyocytes were transfected with siFosl1 and siNC for 48 h, followed by Ki67 (red) and cTnT (green) double staining. Representative images (**a**) and quantification (**b**) of Ki67^+^ cardiomyocytes are shown (*n* = ~60 fields from at least 5 wells per group). Representative *Z*-stack confocal images of Ki67^+^ cardiomyocytes are shown in right panel of **a**. **c**, **d** Proliferation of primary cardiomyocytes were examined using pH3 (red) and cTnT (green) double staining. Representative images (**c**) and quantification (**d**) of pH3^+^ cardiomyocytes are shown (*n* = ~30 fields from at least 5 wells per group). Representative *Z*-stack confocal images of pH3^+^ cardiomyocytes are shown in right panel of **c**. **e**, **f** Proliferation of primary cardiomyocytes were examined using nuclear EdU (red) incorporation assay. Representative images (**e**) and quantification (**f**) of EdU^+^ cardiomyocytes are shown (*n* = ~60 fields from at least 5 wells per group). Representative *Z*-stack confocal images of EdU^+^ cardiomyocytes are shown in right panel of **e**. All data are presented as mean ± SEM. *p < 0.05, ***p < 0.001 (Student’s *t* test).

To further explore whether Fosl1 influences the proliferation of primary CMs, we isolated primary CMs from neonatal mice and examined the cell proliferation with and without Fosl1 silencing. We found that knockdown of endogenous Fosl1 (Supplementary Fig. 9) suppressed the percentage of Ki67^+^ cTnT^+^ cells (Fig. 7a, b), suggesting that Fosl1 is pivotal for the proliferation of primary CMs. This result was further confirmed by double staining with pH3 and Cardiac Troponin T (cTnT; Fig. 7c, d). In addition, nuclear EdU incorporation assay showed that Fosl1 silencing significantly reduced the percentage of EdU^+^ cTnT^+^ cells (Fig. 7e, f).

### Loss of function of Fosl1 suppresses CM proliferation during heart regeneration

**Fig. 8:**
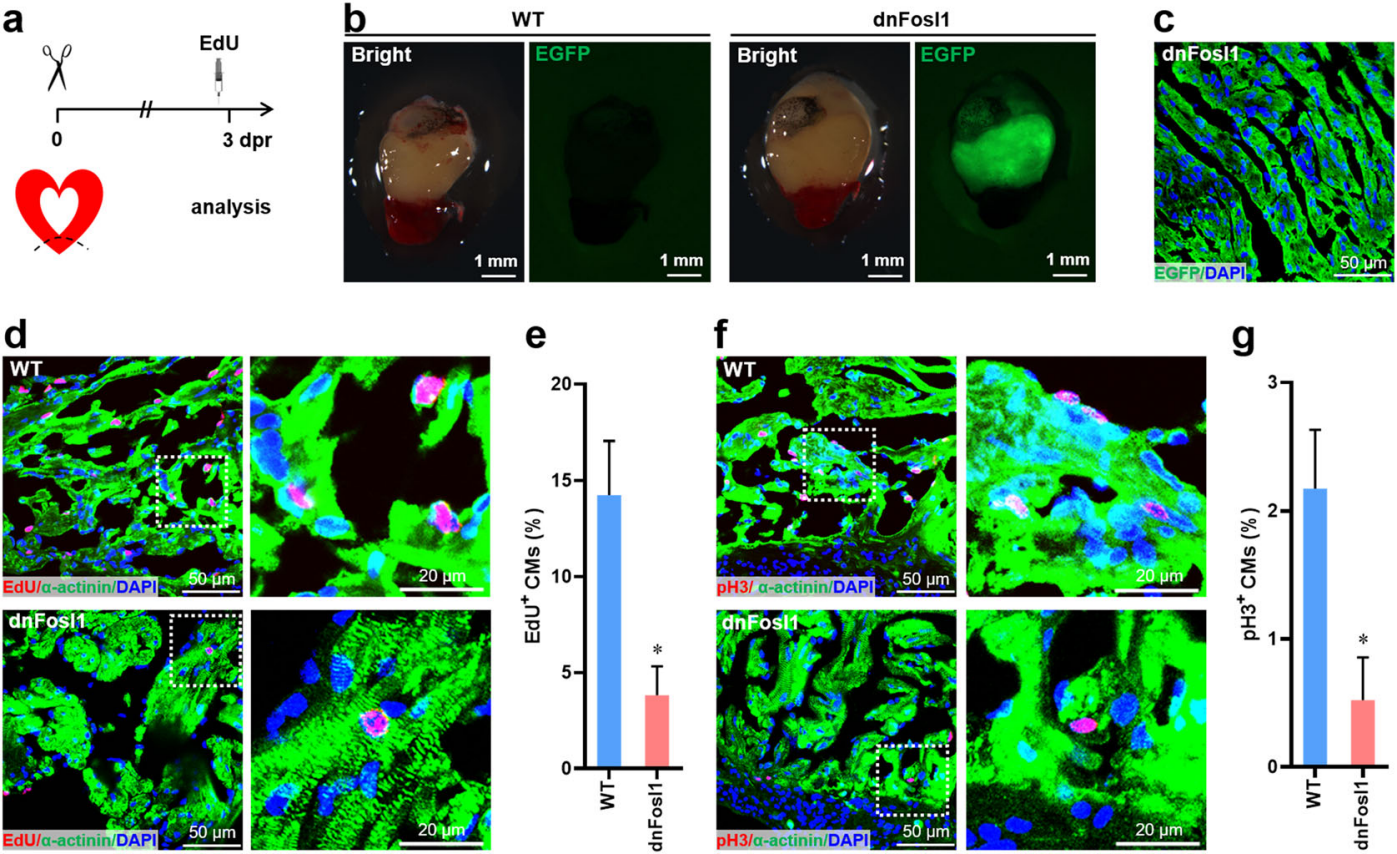
Fosl1 function is required for cardiomyocyte proliferation during *X. tropicalis* heart regeneration. **a** Schematic of heart injury and sample collection in adult *X. tropicalis*. **b** Whole images of hearts isolated from wild-type (WT) and *Tg(Mlc2-dnFosl1-T2A-EGFP)* line at 3 dpr. **c** Validation of EGFP expression in the ventricular section from the dnFosl1 heart. **d**, **e** Representative images (**d**) and quantification (**e**) of EdU^+^ cardiomyocytes in the ventricle apex following EdU injection at 3 dpr (*n* = 5 hearts per group). Right panel of **d** is the magnified confocal images of EdU^+^ cardiomyocytes. **f**, **g** Representative images (**f**) and quantification (**g**) of pH3^+^ cardiomyocytes are shown in the ventricle apex (*n* = 5 hearts per group). Magnified representative confocal images of pH3^+^ cardiomyocytes are shown in right panel of **f**. All data are presented as mean ± SEM. *p < 0.05 versus WT (Student’s *t* test).

To directly determine the in vivo function of Fosl1 during heart regeneration in *X. tropicalis*, a dominant-negative transgenic line was created, which can specifically inhibit Fosl1 function in a CM-specific manner. By truncating *X. tropicalis* Fosl1, we found that Fosl1-NT mutant can be used as dominant-negative Fosl1 (dnFosl1) because it can specifically block the function of Fosl1 instead of other Fos members (Supplementary Fig. 10a–c). Using the dnFosl1, we generated a transgenic *X. tropicalis* line *Tg(Mlc2-dnFosl1-T2A-EGFP)* carrying dnFosl1 under control of CM-specific *Mlc2* promoter (Supplementary Fig. 10d, e). Using this transgenic line and control frogs, we performed cardiac apical resection and analyzed CM proliferation at 3 dpr (Fig. 8a–c). CM proliferation was first evaluated by nuclear incorporation of EdU. The percentage of EdU^+^ α-actinin^+^ cells was significantly decreased in the dnFosl1 hearts compared with controls, indicating that loss of function of Fosl1 inhibits the DNA synthesis and cell cycle entry of CMs during heart regeneration in *X. tropicalis* (Fig. 8d, e). Consistent with these results, pH3/α-actinin double staining revealed that the proliferation of CMs was significantly decreased in the dnFosl1 hearts during regeneration compared with control hearts (Fig. 8f, g). These data indicate that loss of function of Fosl1 leads to a defect in the proliferation of CMs during heart regeneration in *X. tropicalis*.

**Fig. 9:**
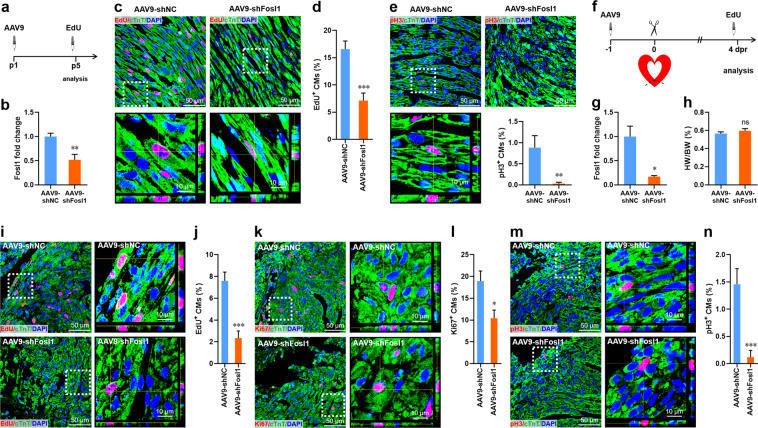
Fosl1 silencing inhibits cardiomyocyte proliferation during heart regeneration in neonatal mouse. **a** Schematic of AAV9-shFosl1 virus injection to silence *Fosl1* in the quiescent neonatal heart. **b** qPCR validation of Fosl1 knockdown in the neonatal heart after AAV9-shFosl1 injection (*n* = 5 hearts). **c**, **d** Representative images (**c**) and quantification (**d**) of EdU^+^ cardiomyocytes in ventricle apex (*n* = 8 hearts). Lower panels of **c** are the magnified confocal images of EdU^+^ cardiomyocytes. **e** Representative images and quantification of pH3^+^ cardiomyocytes in the ventricle apex (*n* = 8 hearts). Lower panels are the magnified images of pH3^+^ cardiomyocytes. **f** Schematic of AAV9-shFosl1 virus injection to silence *Fosl1* in the injured heart at 4 dpr. **g** qPCR validation of Fosl1 knockdown in the injured heart (*n* = 3 hearts). **h** Quantification of heart weight (HW) to body weight (BW) ratio (*n* = 3 hearts). **i**, **j** Representative images (**i**) and quantification (**j**) of EdU^+^ cardiomyocytes in the ventricle apex (*n* = 8 and 5 hearts). Right panels of **i** are the magnified images of EdU^+^ cardiomyocytes. **k**, **l** Representative images (**k**) and quantification (**l**) of Ki67^+^ cardiomyocytes in the ventricle apex (*n* = 8 and 5 hearts). Right panels of **k** are the magnified images of Ki67^+^ cardiomyocytes. **m**, **n** Representative images (**m**) and quantification (**n**) of pH3^+^ cardiomyocytes in the ventricle apex (*n* = 8 and 5 hearts). Right panels of **m** are the magnified images of pH3^+^ cardiomyocytes. All data are presented as mean ± SEM. *p < 0.05, **p < 0.01, ***p < 0.001 versus shNC (Student’s *t* test).

Subsequently, we examined the functional importance of Fosl1 in the neonatal mouse heart. Neonatal mice at postnatal day 1 (p1) were injected with adeno-associated virus 9 (AAV9)-shFosl1 or control (AAV9-shNC) viruses for 4 days, followed by CM proliferation analysis (Fig. 9a). EdU incorporation results revealed that Fosl1 silencing (Fig. 9b) significantly suppressed the cell cycle entry of CMs as evidenced by the decreased percentage of EdU^+^ cTnT^+^ cells (Fig. 9c, d). In line with this result, the percentage of pH3^+^ cTnT^+^ cells was remarkably decreased by the silencing of Fosl1 (Fig. 9e). These results indicate the important role of Fosl1 in neonatal heart growth. To further explore the functional importance of Fosl1 during neonatal heart regeneration, apical resection was performed in the AAV9-shFosl1-injected neonatal mice at p1, followed by CM proliferation assay at 4 dpr (Fig. 9f). Although the silencing of Fosl1 (Fig. 9g) did not influence the HW/BW ratio (Fig. 9h), the proliferation of CMs was greatly suppressed by Fosl1 silencing as demonstrated by the decreased percentages of EdU-, Ki67-, and pH3-positive CMs (Fig. 9i–n). These findings indicate that Fosl1 plays an important role in heart regeneration of neonatal mice.

### Overexpression of Fosl1 improves cardiac function in adult mice upon MI

**Fig. 10:**
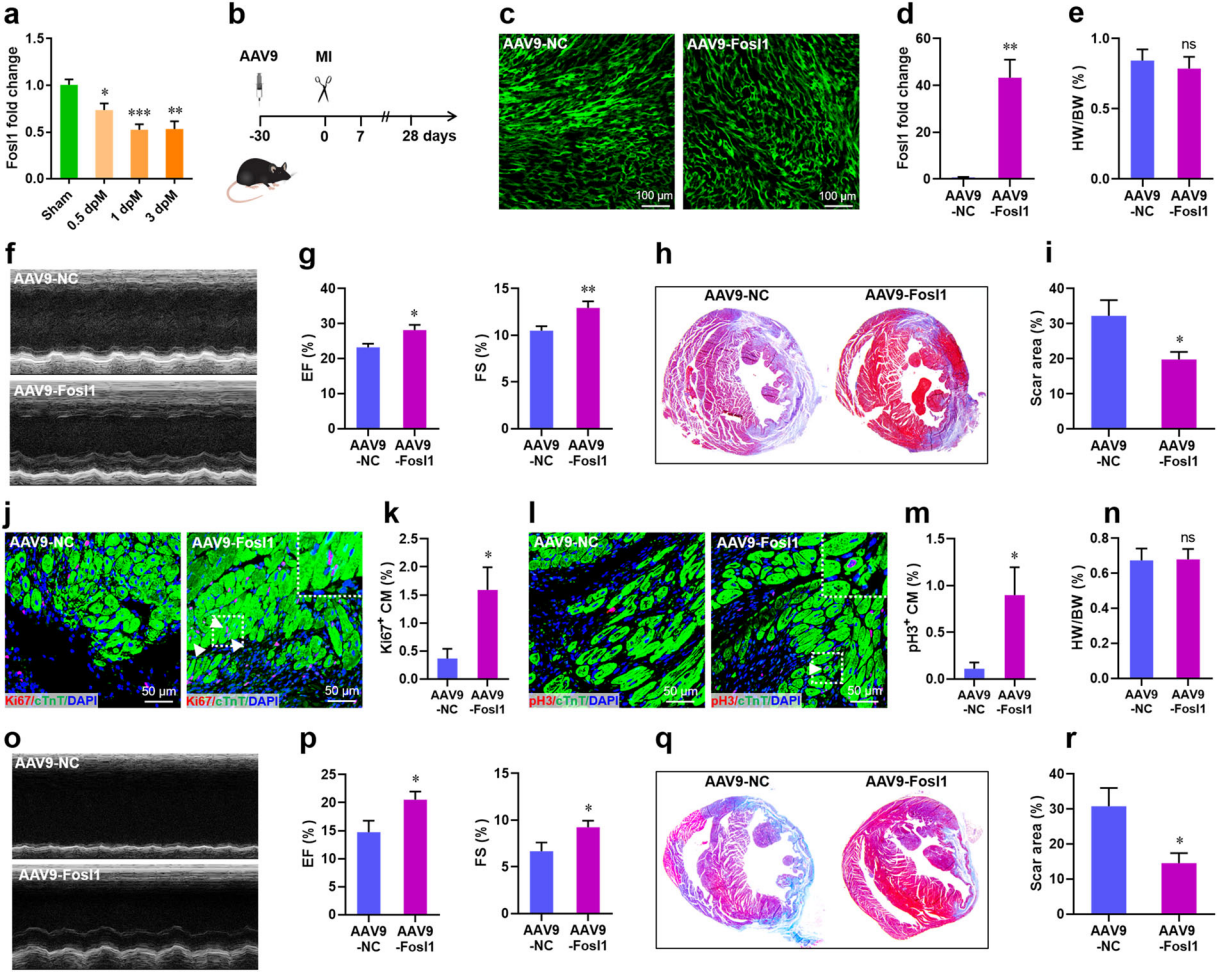
Fosl1 overexpression improves cardiac function in adult mouse upon myocardium infarction. **a** qPCR validation of *Fosl1* expression in the infarcted heart at 0–3 days post-MI (dpM). **b** Schematic of AAV9-Fosl1 virus injection to overexpress *Fosl1* in the infarcted heart. **c** Representative images of EGFP expression in the heart 30 days post AAV9-Fosl1 injection. **d** qPCR validation of *Fosl1* knockdown in the heart 30 days post AAV9-Fosl1 injection (*n* = 3 hearts). **e** Quantification of HW to BW ratio at 7 dpM (*n* = 7 hearts). **f**, **g** Representative images of M-model echocardiography (**f**) and quantification of LVEF (**g**, left) and LVFS (**g**, right) at 7 dpM are shown (*n* = 11 hearts). **h**, **i** Representative images (h) and quantification (**i**) of scar size at 7 dpM (*n* = 5 hearts). **j**, **k** Representative images (**j**) and quantification (**k**) of Ki67^+^ cardiomyocytes at 7 dpM (*n* = 5 hearts). Right upper panels of **j** are the magnified images of Ki67^+^ cardiomyocytes. **l**, **m** Representative images (**l**) and quantification (**m**) of pH3^+^ cardiomyocytes at 7 dpM (*n* = 5 hearts). Right upper panels of **l** are the magnified image of pH3^+^ cardiomyocytes. **n** Quantification of HW to BW ratio at 28 dpM (*n* = 7 hearts). **o**, **p** Representative images of M-model echocardiography (**o**) and quantification of LVEF (**p**, left) and LVFS (**p**, right) at 28 dpM are shown (*n* = 10 hearts). **q**, **r** Representative images (**q**) and quantification (**r**) of scar size at 28 dpM (*n* = 5 hearts). All data are presented as mean ± SEM. *p < 0.05, **p < 0.01, ***p < 0.001 versus control (Student’s *t* test).

To further determine whether Fosl1 is related to heart repair in non-regenerative model, we checked the expression patterns of Fosl1 during MI in adult mice without capacity to regenerate the injured heart. Contrary to the great upregulation of Fosl1 during *X. tropicalis* heart regeneration, Fosl1 expression was significantly decreased in the infarcted hearts from 0 to 3 days post-MI (dpM) compared with the sham-operated hearts (Fig. 10a). These different cardiac expression patterns of Fosl1 in adult *X. tropicalis* and mouse after heart injury are likely to reflect the contrary capacities of heart regeneration in these two adult species. To further determine whether Fosl1 overexpression improves heart repair after MI, adult mice were injected with AAV9-cTnT-Fosl1 (AAV9-Fosl1) for 30 days to overexpress Fosl1 in the heart (Fig. 10b–d). The injected mice were subjected to MI surgery, followed by cardiac function analysis. At 7 dpM, no significant difference in HW/BW ratio was detected between Fosl1-overexpressed hearts and controls (Fig. 10e). However, Fosl1 overexpression significantly promoted the cardiac function as evidenced by the increased left ventricular ejection fraction (LVEF) and left ventricular fractional shortening (LVFS) values (Fig. 10f, g) and the decreased scar size (Fig. 10h, i). In consistent with these results, the percentages of Ki67^+^ and pH3^+^ CMs were also increased by Fosl1 overexpression, indicating the elevated levels of CM proliferation during heart injury (Fig. 10j–m). To further determine the long-term effects, cardiac function was also evaluated at 28 dpM. Although there was no significant difference in the HW/BW ratio (Fig. 10n), increased LVEF and LVFS levels (Fig. 10o, p) and decreased scar size (Fig. 10q, r) were detected in the Fosl-overexpressing hearts compared with controls, suggesting that Fosl1 has a long-term effect on the improvement of cardiac function after injury. Taken together, these additional experiments further confirm that Fosl1 indeed has a beneficial effect on heart repairs in non-regenerative contexts.

## Discussion

It is important and promising to investigate cardiac regenerative potential and understand the underlying mechanism in model organisms for the therapy of damaged heart in human^41^. At present, most data of heart regeneration are obtained from zebrafish and neonatal mouse models^10–14,42,43^. In fact, urodela including newt and salamander, which is evolutionarily between zebrafish and mouse, are also demonstrated to generate injured heart even in adulthood^16,17^. Recently, our group reported the heart regenerative potential of adult *X. tropicalis*, an anuran amphibian model with a real diploid genome and more close evolutionary relationship with mammalians, through histological analysis^26^. However, the underlying molecular and cellular mechanism remains largely unclear. In the present study, we further systematically analyzed the molecular and cellular mechanisms that control heart regeneration in adult *X. tropicalis*. We demonstrated that the heart of adult *X. tropicalis* aged 6 months could regenerate upon ventricular apex resection, mainly through the proliferation of CMs. Moreover, our data also demonstrated that Fosl1 plays an important role in promoting CM proliferation and heart regeneration, at least in part, through interaction with JunB and promoting the expression of cell cycle regulators, including ccnt1.

Previous studies have clearly demonstrated that adult teleost fish (zebrafish) and urodele amphibians (newt and salamander) can completely regenerate injured heart without scarring through CM proliferation, suggesting that zebrafish and urodele amphibians retain a remarkable capacity for heart regeneration throughout life^12–14,16,17,42,43^. Anuran amphibians, including *X. tropicalis* and *X. laevis*, retain powerful capacity to regenerate spinal cord, tail, eye, and limb^20–23^. We previously have reported that adult *X. tropicalis* at age 1 year can regenerate the injured heart^26^. However, 1 year is a long time and a large disadvantage for research work. Using 1-year-old *X. tropicalis* for research work will greatly increase the time costs. Given that *X. tropicalis* grows to adult in 4 months^27^, the 6-month-old *X. tropicalis* used in this study is really in adult stage. Importantly, 6-month-old *X. tropicalis* will save a half year time compared with the 1-year-old animal used in our previous study^26^. On the other hand, a recent study has demonstrated that 6-month-old juvenile *X. laevis* permanently lose cardiac regenerative capacity^24^, which also prompts us to ask whether 6-month-old *X. tropicalis* can regenerate the heart following injury. In consistent with our previous study, histological analysis revealed the powerful regenerative potential of heart after apex resection in 6-month-old *X. tropicalis* (Fig. 1 and Supplementary Figs. 1–3). Moreover, our findings demonstrated that the proliferation of CMs plays important roles in *X. tropicalis* heart regeneration (Figs. 2 and 3 and Supplementary Figs. 4–6), which is consistent with the mechanisms for heart regeneration in adult zebrafish and neonatal mouse^10,12^.

In the present study, RNA-seq and qPCR analysis revealed that substantial proliferation-related genes were significantly upregulated at the early stage of apex resection (Figs. 4 and 5). Among these genes validated by qPCR, fosl1 showed the maximum changes (>80-fold), indicating that fosl1 might be the most potential target involved in heart injury and regeneration in *X. tropicalis*. Importantly, overexpression of *X. tropicalis* Fosl1 (xFosl1) increases the proliferation of mammalian CM cell line (H9c2) (Fig. 6a–d). Moreover, knockdown of the endogenous Fosl1 significantly inhibits the proliferation of H9c2 cells (Fig. 6e–l) and primary CMs isolated from neonatal mouse heart (Fig. 7). These data strongly indicate that Fosl1 might be involved in CM proliferation and heart regeneration in amphibians and mammals. Fosl1 was originally considered a gene involved in controlling G1/S phase transition by upregulating cyclin D1 in mammals^44^, suggesting the critical role of Fosl1 in cell cycle regulation. This idea was partially supported by our models revealing that cyclins, cyclin-dependent kinases, and checkpoint kinases were increased during *X. tropicalis* heart regeneration at 0.5 dpr (Figs. 4j–m and 5g), which was accompanied with remarkable upregulation of *fosl1* (Fig. 5a, b). In addition, luciferase reporter and ChIP-PCR assay further approved that ccnt1 might be direct regulated by Fosl1 during heart regeneration (Fig. 5h–m). Taken together, our data suggest that transcription factor Fosl1 might promote CM proliferation by upregulating the expression of cell cycle regulators including Ccnt1, thereby improving heart regeneration in vertebrates.

Recently, Beisaw and colleagues revealed that AP-1 transcription factor function in CMs is necessary for zebrafish heart regeneration. They found that AP-1 plays important roles in CM dedifferentiation and proliferation, sarcomere disassembly, CM protrusion as well as CM chromatin landscape after injury^33^. They also found that in vitro overexpression of Fosl1 and JunB leads to changes in behavior of primary rat CMs^33^. In vitro and in vivo data from Beisaw’s work suggest that AP-1 transcription factor function is necessary for zebrafish heart regeneration. These findings are in agreement with previous reports showing that AP-1 transcription factors are involved in cardiovascular diseases, such as heart failure and MI^30–32^.

It is well known that Fos family including four members (c-Fos, FosB, Fosl1, and Fosl2) acts by forming heterodimeric complexes with Jun proteins (c-Jun, JunB, JunD), which control gene expression through interaction with the AP-1 DNA consensus element^38,39,44–46^. Beisaw and colleagues suggested the functional importance of AP-1 during zebrafish heart regeneration by inhibiting AP-1 function using a dominant-negative (A-Fos) transgenic line^33^. The previously reported dominant-negative A-Fos can efficiently inhibit the function of AP-1 transcription factor by interfering with most members of bZIP family proteins^47^. However, each bZIP member has a distinct role under different conditions^48,49^. Therefore, it is difficult to identify the unique AP-1 member that is critical for heart regeneration using the A-Fos transgenic line. Importantly, whether and how Fosl1 regulates heart regeneration in vivo remains unclear. To solve this issue, we created a dnFosl1 that can specifically inhibit the function of Fosl1 instead of other Fos members (Supplementary Fig. 10). Taking use of the dnFosl1, we generated a transgenic *X. tropicalis* line *Tg(Mlc2-dnFosl1-T2A-EGFP)* carrying the dnFosl1 under control of Mlc2 promoter. Using this transgenic line and control frogs, we further demonstrated that loss of function of Fosl1 inhibits the proliferation of CMs during heart regeneration in *X. tropicalis* (Fig. 8). The functional importance of Fosl1 during heart regeneration was also confirmed in neonatal mice model (Fig. 9). Importantly, Fosl1 overexpression was demonstrated to improve the cardiac function in adult mouse upon MI (Fig. 10). Taken together, these in vivo loss- and gain-of-function approaches substantially revealed the important role of Fosl1 in heart regeneration of vertebrates.

In conclusion, our results revealed that the cardiac regeneration of adult *X. tropicalis* appears to mainly result from CM proliferation in the injured heart. Using loss- and gain-of-function approaches, we demonstrated that Fosl1 plays an important role in CM proliferation and heart regeneration in vertebrates, at least in part, through interaction with JunB, thereby promoting the expression of cell cycle regulators, including Ccnt1. Our data proposed the deeper understanding of the mechanism by which Fosl1 regulates heart regeneration and might provide a new insight into the regenerative response in adult vertebrates. It should be noted that CM labeling was mostly performed using whole-cell staining of CMs in the present study. Although rigorous quantification method was used to minimize the potential interference of proliferating nCMs, most strict methods, such as cell fate tracing technology and nuclear staining of CMs, should be used to support our conclusion in the future.

## Methods

### Animals

Western clawed frogs (*X. tropicalis*) at 6–7 months (males) were used in this study, considering that *X. tropicalis* has a much shorter life cycle and grows to adult in 4 months^27^. Thus, the *X. tropicalis* at 6 months old are really in adult stage. Apical resection surgeries in frogs were performed as described previously^12,26^, with some modification. In brief, frogs were anesthetized by cooling in ice for about 1–2 min. The anaesthetized frogs were moved to plate with ventral side up, fixed by thumbtacks into limbs. A small incision penetrating the skin and pericardial sac was induced by iridectomy scissors. The ventricle was exposed by gentle pressure, and ventricle apex (~14% of whole ventricle) was then removed by scissors. The incision was blotted by gossypium asepticum prior to quickly clotting of wounds. Body wall incisions were sutured, and frogs were waked up within several minutes in culture dish with litter water, which does not submerge the body. Surgical frogs usually began to wake up within 10 min. This surgical procedure permits over 90% frogs to survive, with all deaths occurring on the day of surgery. After waking up, frogs were returned to water and stimulated to breathe by vigorously squirting water with a pipette. Sham operation was performed as the above procedure without ventricle resection. To further demonstrate surgical reproducibility, 29 resected and 16 intact ventricles were immediately weighed after surgery. The hearts were collected after surgery and briefly rinsed in phosphate-buffered saline (PBS) solution to remove residual blood from the ventricles. The ventricles without non-cardiac tissue and atria were blotted and weighed. Our group has found that this surgical procedure is highly reproducible.

Apical resection in neonatal mice (C57BL/6) were performed as previously described^10^, followed by heart collection and analysis at the indicated time points as described below. In brief, lateral thoracotomy was performed by blunt dissection of the intercostal muscles following skin incision. After exposing the left ventricular chamber, iridectomy scissors were used to resect the apex of neonatal hearts. Following apical resection, neonates were removed from the ice bed, thoracic wall incisions were sutured with 7-0 non-absorbable silk suture, and the skin wound closed using skin adhesive. Sham-operated mice underwent the same procedure without apical resection. Neonates were then placed under a heat lamp and warmed for several minutes until recovery. MI in adult mice were induced by permanent ligation of the left anterior descending artery (LAD) as previously described^50^. In brief, the left thoracic region was shaved and sterilized. The heart was exposed through a left thoracotomy after intubation. The LAD was permanently ligated using a suture. The thoracotomy and skin were then sutured closed in layers. After removal of the excess air from the thoracic cavity, the mouse was removed from ventilation when normal breathing was established. For some animal experiments, AAV9 viruses were injected before heart injury at the indicated time points as indicated in the related schematics. After injection with AAV9 viruses and MI performance, the cardiac function was evaluated at the indicated time points using the Vevo® 2100 ultrasound system (Visualsonics, Toronto, Canada) equipped with a high-frequency (30 MHz) linear array transducer. Animal experiment protocols were approved by the Institutional Animal Care and Use Committee of Jinan University.

### AAV9 production

The mouse Fosl1 coding sequences were amplified and cloned into AAV serotype-9 expressing plasmid to overexpress Fosl1 under the control of the cardiac-specific cTNT promoter (AAV9-cTnT-Fosl1, also termed as AAV9-Fosl1). AAV9-NC (virus packaged with empty plasmid) served as control for AAV9-Fosl1. In addition, Fosl1-specific short hairpin RNA (shRNA) or negative control (RiboBio, Guangzhou, China) were cloned into AAV9-shRNA-expressing plasmid to generate AAV9-shFosl1 or AAV9-shNC plasmids, respectively. AAV9 viruses were packaged and produced using the AAV Helper-Free System (DongBio.Co.Ltd, Shenzhen, China). For AAV9-shFosl1 virus delivery in vivo, viruses were subcutaneously injected into neonatal mice at a dose of 2 × 10^11^ V.G./mouse at p1. The AAV9-Fosl1 viruses were delivered into adult mice at a dose of 1 × 10^11^ V.G./mouse by tail-vein injection. MI was performed 30 days post-injection, followed by cardiac function assay. The schematic of AAV9 virus injection can be found in Figs. 9 and 10.

### Histology

Hearts were fixed in 4% paraformaldehyde overnight at room temperature. The following day, hearts were moved to a graded series of ethanol (70, 80, 95, 100%) followed by paraffin embedding. Paraffin sections (4 μm) were cut through the entire ventricle. Hematoxylin/eosin (H&E) and Masson’s trichrome staining were performed according to the standard procedures. H&E-stained sections were used to quantify muscle replacement during cardiac regeneration. The largest three ventricular sections from each animal were quantified using Image-Pro Plus version 6.0 (Media Cybernetics, Inc., Rockville, MD, USA). The mean area of resected ventricles was expressed as a percentage difference relative to sham-operated ventricles (set to 100%). CR and IR of the resected apex were evaluated by the largest ventricular section of each heart. CR heart apex was characterized by the intact ventricular wall but not for IR heart. In certain experiments of Masson’s trichrome staining, the largest three sections from each heart were used to quantify cardiac fibrosis using the Image-Pro Plus version 6.0 software and then expressed as a percentage relative to the control group.

### Wheat germ agglutinin (WGA) staining

Sections were deparaffinized, rinsed three times in PBS, and fixed in 4% paraformaldehyde for 30 min at 37 °C. After washing three times in PBS, sections were then incubated for 10 min at room temperature with primary antibody against WGA conjugated to Alexa Fluor 488 (5 μg/ml, ThermoFisher, W11261). Sections were rinsed three time in PBS and mounted in Antifade Mounting Medium (Jackson ImmunoResearch Laboratories). To quantify the cell size, four independent hearts (9–13 sections) per group were captured with laser-scanning confocal microscope (LSM 700, Zeiss). Image-Pro Plus version 6.0 software was used to quantify the size of each cell.

### Immunofluorescence

Hearts were fixed in 4% paraformaldehyde overnight at room temperature and embedded in paraffin as described above. Immunofluorescence staining was performed on the paraffin sections (4 μm). For antigen retrieval, sections were boiled for 15 min in citrate antigen retrieval buffer (Abcam, USA), washed three times in PBS, and blocked in 5% goat serum for 1 h at room temperature. Sections were then incubated overnight at 4 °C with primary antibodies against Ki67 (Abcam, ab16667, 1:250), phosphohistone H3 (pH3) (CST, #3377, 1:800 dilution), α-Actinin (Sigma, SAB3300072, 1:200 dilution), and cTnT (ThermoFisher, MA512960, 1:200), or with primary antibodies against PCNA (Sigma, MABE288, 1:1000 dilution) and Mef2C (Abcam, ab227085, 1:100 dilution), respectively. Antibody against Fosl1 (Abcam, ab252421, 1:500 dilution) was used to detect Fosl1 expression during heart regeneration in neonatal mouse. After washing three time in PBS, the sections were incubated with anti-mouse secondary antibody conjugated to Alexa Fluor 488 (Jackson Immuno Research) and with anti-rabbit secondary antibody conjugated to Cy3 (Proteintech Group, USA) for 1 h at room temperature to identify pH3^+^ α-Actinin^+^ CMs or PCNA^+^ Mef2C^+^ CMs. Sections were then rinsed three times in PBS, stained with 4,6-diamidino-2-phenylindole (DAPI; Sigma) at a concentration of 2 μg/ml to label nucleus, and mounted with Antifade Mounting Medium (Jackson ImmunoResearch Laboratories). Images were captured by laser-scanning confocal microscope (LSM 700, Zeiss) and analyzed by the ZEN 2012 software (Zeiss). To minimize the potential interference of proliferating nCMs during whole-cell staining of CMs, only the proliferating nuclei completely surrounded by whole-cell staining marker of CMs were counted in our experiments. The proliferating nuclei without or partially surrounded by whole-cell staining marker of CMs were excluded. This strict quantification method would decrease the potential interference of proliferating nCMs as far as possible.

### EdU labeling

For EdU-labeling experiments, animals were injected intraperitoneally with 50 μl of a 2 mg/ml solution of EdU (RiboBio, Guangzhou, China) dissolved in sterile water. Hearts were embedded in Tissue-Tek optimal cutting temperature compound (Sakura, USA) for frozen section (4 μm). Sections were rinsed three times in PBS and fixed in 4% paraformaldehyde for 30 min. After rinsing three times again, citrate antigen retrieval was performed as described above. Sections were then incubated with 2 mg/ml glycine solution for 10 min, permeabilized with 0.5% Triton X-100 in PBS for 10 min, and then rinsed with PBS once for 5 min. This was followed by incubation with Apollo®488 staining solution (1×) at room temperature for 30 min. Permeabilization was performed again with 0.5% Triton X-100 in PBS twice for 10 min. Sections were then rinsed with methanol for 5 min, washed with PBS once for 5 min, blocked with 5% goat serum for 1 h, and followed by incubation with primary antibody against α-Actinin overnight. The following day, incubation with anti-mouse secondary antibody conjugated to Cy3 was performed. Sections were washed three times in PBS, stained with DAPI for 10 min to label nuclei, and mounted in Antifade Mounting Medium. Images were captured by laser-scanning confocal microscope (LSM 700, Zeiss) and analyzed by the ZEN 2012 software (Zeiss).

To analyze CMs proliferation at multiple time points, EdU was injected three times within 48 h followed by heart collection immediately. The last one out of three injections were performed 8 h prior to heart collection. Sham-operated frogs were injected with EdU prior to heart resection surgery, followed by heart collection at 0 dpr. For EdU pulse-chase experiments, EdU was injected once every 3 days for 30 days to label all proliferating CMs during the whole period of cardiac regeneration. The last injection was performed 8 h prior to heart collection at 30 dpr. Sham-operated frogs underwent the same procedure without the apical resection. As mentioned above, to minimize the potential interference of proliferating nCMs during whole-cell staining of CMs, only the proliferating nuclei completely surrounded by whole-cell staining marker of CMs were counted in our experiments. The proliferating nuclei without or partially surrounded by whole-cell staining marker of CMs were excluded.

### Visualization of regenerated apex and systolic function quantification

Both sham-operated and resected frogs at 7, 14, and 30 dpr were anesthetized in ice for 2 min. Hearts were quickly extracted and cleaned to remove blood clot. Cleaning hearts were then cultured in the medium (Dulbecco’s Modified Eagle’s Medium (DMEM) plus 10% (vol/vol) fetal bovine serum (FBS), 1% (vol/vol) Minimum Essential Medium–non-essential amino acids, 100 U/ml penicillin, 100 μg/ml streptomycin, and 50 μM 2-mercaptoethanol) as described previously^51^. Images of regenerated apex and videos of beating heart were captured using Leica M205FA stereo fluorescence microscope. To analyze heat beating frequency, videos were captured within 1 min for each heart to quantify cardiac systolic function during regeneration. The beating frequency of the cultured hearts was calculated by the beating number per 20 s. Video capture of each cultured heart was finished as soon as possible within 30 min. Whole images of the cultured hearts were captured to visualize apex regeneration at different time points.

### RNA-seq and bioinformatics analysis

For total RNA isolation, ventricles of *X. tropicalis* frogs were extracted in the sham-operated group and resected groups at 0.5–60 dpr, respectively. RNA preparation, library construction, and sequencing on GBISEQ-500 platform was performed as previously described^52^. After filtering the reads with low quality, clean reads were then obtained and mapped to the reference genome of *X. tropicalis* (version 9.1) with HISAT^53^. Gene expression level was quantified by a software package called RSEM^54^ and expressed as fragments per kilobase of exon per million fragments mapped (FPKM). Differentially expressed genes were detected using NOISeq method^55^ with probability ≥0.8 and fold change (FC) of FPKM ≥ 2. GO analysis was performed using online tool DAVID 6.8 (https://david.ncifcrf.gov/summary.jsp), and terms with *p* value ≤0.05 were included. Differentially expressed gene heat maps were clustered by hierarchical clustering using the cluster software^56^. RNA-seq data are available from NCBI Sequence Read Archive (BioProject accession: PRJNA401530). GSEA was performed to identify gene sets from signaling pathways that showed statistical differences between two groups by using the GSEA software (http://software.broadinstitute.org/gsea/index.jsp)^57,58^.

### Cell culture and transfection

H9c2 cells purchased from ATCC (Manassas, VA, USA) were cultured in DMEM containing 10% (v/v) FBS, 100 U/ml penicillin, and 100 µg/ml streptomycin (all from Gibco, Carlsbad, CA, USA), at 37 °C in a 5% CO_2_ incubator. Culture medium was replaced every 2–3 days. To knock down the endogenous expression of rat *Fosl1* genes in H9c2 cells, siRNAs (RiboBio, Guangzhou, China) were transfected into cells with Lipofectamine 3000 (Life Technologies) according to the manufacturer’s instructions. siRNA sequences used in this study are listed in Supplementary Table 1. Frog *fosl1* and *junb* were isolated from a cardiac cDNA library of *X. tropicalis* and subcloned into the pcDNA3.1 vector to construct pcDNA-Fosl1, pcDNA-Fosl1-Flag, and pcDNA-JunB-HA plasmids. H9c2 cells were transfected with pcDNA-Fosl1 to overexpress frog *fosl1* and analyze cell survival and proliferation. Primary CMs were, respectively, isolated from neonatal mice^35^ and adult *X. tropicalis*^59^ as previously described. During the process of CM isolation, all washing solution of CM pellets were collected and centrifuged at higher speed (500 × *g*) to collect nCMs for RNA extraction and qPCR analysis.

### Stable knockdown cell line

H9c2 *Fosl1* knockdown cell line was generated using LentiCRISPRv2 system as described previously^60^. In brief, single-guide RNA (sgRNA) sequences were cloned into the LentiCRISPRv2 plasmid and co-transfected with viral packaging plasmids (psRAX2 and pMD2G) into HEK293T cells. H9c2 cells were infected with the filtered viral supernatants and selected with 1 mg/ml puromycin for 1 week when control cells were completely killed. After puromycin screening, the remain living cells were used to establish single-cell colonies by serial dilution. Finally, five single-cell colonies with four genotypes were generated and confirmed by DNA sequencing (Supplementary Fig. 8). Among them, the single-cell colony deleting 4 base pairs (Δ4), which can induce frameshift mutant, was further used for cell counting assay. The sgRNA sequence designed to target Exon 2 of rat Fosl1 was as follows: 5’-GCT CGT ATG ACT CCT GGT CG-3’.

### Quantitative real-time PCR

Total RNA was isolated from resected and sham-operated ventricles at 0.5, 3, and 7 dpr (*n* = 4 hearts per group) or from H9c2 cells treated as below, using TRI Reagent (Molecular Research Center Inc., USA). cDNA was synthesized from total RNA (2 μg) and oligo (dT)18 primers (0.5 μg) using the ReverTra Ace® qPCR RT Kit (Toyobo, Japan). The qPCR analysis was performed using QuantiTect SYBR green PCR Master Mix (Qiagen GmbH, Hilden, Germany) and the MiniOpticon Real-Time PCR System (Bio-Rad, CA, USA). After denaturation for 10 min at 95 °C, the reactions were subjected to 45 cycles of 95 °C for 30 s, 60 °C for 30 s, and 72 °C for 30 s. GAPDH was used as the internal standard control to normalize gene expression using the ΔΔCt method. Primer sequences are listed in Supplementary Tables 2–4.

### Analysis of cell proliferation in vitro

H9c2 cells were plated in 24-well trays at a concentration of 1 × 10^4^ cells per well and incubated overnight, followed by transfection with siRNA or pcDNA-xtFosl1 plasmid for 24–48 h. Transfected cells were treated with 10 μM EdU solution for 2 h to label proliferating cells using the Cell-Light™ EdU Apollo®567 In Vitro Imaging Kit (RiboBio, China), according to the manufacturer’s instructions. Images were captured by laser-scanning confocal microscope (LSM 700, Zeiss) and analyzed by the ZEN 2012 software (Zeiss). A total of about 10 fields were randomly selected per well, and the number of proliferating cells were expressed as a percentage of the total number of cells scored. In addition, cell viability was analyzed using the CCK-8 Cell Counting Kit (Beyotime Biotechnology, China) according to the manufacturer’s instructions, as previously described.

### CoIP assay

Potential interaction between *X. tropicalis* Fosl1 (xFosl1) and xJunB proteins was examined by CoIP assay in *X. tropicalis* embryos. CoIP was performed by using the Pierce™ Classic Magnetic IP/Co-IP Kit (#88804, Thermo Scientific). The Flag-tagged xFosl1 and HA-tagged xJunB plasmids were co-injected into the animal pole of *X. tropicalis* embryos (fertilized eggs) at one-cell stage, using microinjector (PV-820, WPI). Each embryo was injected with 2 nl solution containing both plasmids (100 pg per plasmid). On the next day, injected embryos were inspected, and abnormal ones were then removed. Proteins were extracted from the normally developed embryos 48 h after injection, by using ice-cold IP lysis/wash buffer supplemented with protease inhibitor. The mixture was incubated on ice for 5 min with periodic mixing and centrifuged at 13,000 × *g* for 10 min to gain the supernatant. Second, the supernatant (containing 8 μg protein) was incubated overnight with anti-Flag (Sigma, F1804) or anti-HA (CST, #3724) at 4 °C on a gentle shaker. Magnetic bead-conjugated mouse IgG (CST, #5873) or normal rabbit IgG (CST, #2729) was used as negative control. Third, 25 µl magnetic beads were washed by using 175 µl IP lysis/wash buffer gently and washed by using 1 ml IP lysis/wash buffer again after discarding the supernatant. Magnetic beads and IP sample were mixed gently and incubated at room temperature on slow rotation for 1 h. Beads were collected with magnetic stand, and the supernatant was removed. Five hundred microliters of IP lysis/wash buffer was added to the tube and gently mixed and the supernatant was discarded twice. Five hundred microliters of water was added to the tube and gently mixed. Finally, the antigen/antibody complex was eluted by elution buffer and analyzed by western blotting after denaturation at 100 °C for 7 min. To calculate the relative expression of xFosl1-Flag and xJunB-HA in embryos, β-actin serves as a reference for the sample loading. In addition, the interaction between Fosl1 and JunB during neonatal mouse heart regeneration was also determined by CoIP assay as described above using anti-Fosl1 (Abcam, ab252421) and anti-JunB (Abcam, ab128878) antibodies. All blots are derived from the same experiment and were processed in parallel. The uncropped blots are listed in Supplementary Fig. 11.

### Luciferase reporter assay

The promoter sequences (−2000 to −1 bp, upstream of transcription start site) of the *X. tropicalis* target genes (Table S5) were analyzed by the JASPAR 2018 online software (http://jaspar.genereg.net/)^61^ to determine potential FOSL1::JUNB-binding sites. The predicted binding site with highest score for each promoter was further analyzed by *Gaussia* luciferase reporter assay to evaluate the in vitro binding and regulating effects of *X. tropicalis* Fosl1 on target genes. Briefly, to construct the reporter plasmids, promoter regions of target genes comprising predicted FOSL1::JUNB-binding sites were amplified and cloned into the pMCS-*Gaussia* Luc plasmids. HEK293T cells were co-transfected with reporter plasmid and pcDNA-Fosl1 plasmid (100 ng for each plasmid) using LipoFiter Liposomal Transfection Reagent (Hanbio Biotechnology). pTK-Red Firefly Luc plasmid (10 ng) was used as luciferase control. Two days after transfection, luciferase reporter assay was carried out using the Pierce^TM^ *Gaussia*-Firefly Luciferase Dual Assay Kit (ThermoFisher Scientific) according to the manufacturer’s protocol. Relative luciferase activity was measured using a BioTek Synergy^TM^ 4 multimode microplate reader (BioTek Instruments). The activity of the *Gaussia* luciferase was normalized with that of Firefly luciferase. To further verify the potential binding sites, mutant luciferase reporter plasmids were generated by the KOD-Plus-Mutagenesis Kit (Toyobo, Osaka, Japan), according to the manufacturer’s protocol.

### ChIP assay

The pcDNA-xFosl1-Flag plasmid (200 pg) was injected into the animal pole of *X. tropicalis* embryos (fertilized eggs) at one-cell stage. Genome DNA was isolated 48 h after injection and subjected to ChIP assay using the SimpleChIP® Plus Enzymatic Chromatin IP Kit (CST, #9004). Flag antibody (Sigma, F1804) was used. Normal goat IgG (CST, #2729) was used as a control. The DNA isolated from input chromatin fragments and from the precipitated chromatin fragments by anti-Flag antibody or control IgG was subjected to PCR using primers flanking the consensus Fosl1-binding sites on *ccnt1* promoter. PCR products were determined on a 1.5% agarose gel. Relative binding ability of Fosl1 was expressed as the DNA signals relative to input. ChIP-PCR primers used in this study are as follows: ccnt1-ChIP-F: 5’-GCC CAT GTG ACC CTT CAA GA-3’ and ccnt1-ChIP-R: 5’-TGT AGT CTC TTC AAG CAA TAG TT-3’. In addition, ChIP-qPCR was performed using genome DNA isolated from regenerating neonatal mouse heart to determine the interaction between Fosl1 protein and Ccnt1 promoters. Fosl1 antibody (Abcam, ab252421) was used to precipitate genome DNA fragments. Normal goat IgG antibody (CST, #2729) was used as a control. ChIP-qPCR primers used in this study are as follows: Ccnt1-ChIP-F: 5’-GAA CAT CTC TAA CTC TGC TC-3’ and Ccnt1-ChIP-R: 5’-ATC AAC ATT ACT TTC AGA GT-3’.

### Construction of dnFosl1 and transgenic *X. tropicalis* line

To create a dnFosl1, *X. tropicalis* Fosl1 protein was mutated and truncated to generate several mutant polypeptides. The basic leucine zipper (bZIP) and C-terminal domains were removed from Fosl1 full-length protein (298 amino acids (aa)) to generate N-terminal domain alone (aa 1–123, termed as Fosl1-NT). Fosl1 full length was removed from the DNA-binding (DB) domain (aa 124–147) or leucine zipper (LZ) domain (aa 149–177) to generate Fosl1∆DB or Fosl1∆LZ mutant polypeptides, respectively. The relative DNA sequences of these three mutant polypeptides were cloned into pcDNA3.1 plasmid to generate pcDNA-Fosl1-NT, pcDNA-Fosl1∆DB, and pcDNA-Fosl1∆LZ plasmids, respectively. The previously reported dominant-negative A-Fos was used as the positive control in this study^47^. Full length of Fos, FosB, and Fosl2 were amplified from *X. tropicalis* cDNA library and cloned into pcDNA3.1 plasmid to construct pcDNA-Fos, pcDNA-FosB, and pcDNA-Fosl2 plasmids. Seven AP-1 consensus DNA-binding elements (TGAGTCA) were cloned into the pMCS-*Gaussia* Luc plasmid to construct the reporter plasmid pAP1-GLuc. HEK293T cells were co-transfected with reporter plasmid and pcDNA-Fosl1 with or without mutants. pTK-Red Firefly Luc plasmid was used as luciferase control. Luciferase reporter assay was then performed and calculated as described above. Fosl1-NT with the maximum capacity to inhibit Fosl1-driven luciferase activity was used as the dnFosl1 due to its safety for other Fos proteins (Supplementary Fig. 10a–c). The DNA fragment of dnFosl1 was then fused with a T2A-EGFP coding sequence and cloned into a I-SceI transgenesis vector downstream of the 3 kb *mlc2* promoter to construct the transgenesis plasmid pMlc2-dnFosl1-T2A-EGFP. The transgenesis plasmid and I-Sce1 enzyme were then injected into the animal pole of *X. tropicalis* embryos at one-cell stage to generate a transgenic line. Founder *X. tropicalis* were identified and propagated to establish *Tg (Mlc2-dnFosl1-T2A-EGFP)* stable line.

### In situ hybridization

Heart with or without apical resection injury was isolated from *X. tropicalis* during regeneration at 0.5 dpr. Fosl1 expression in heart tissue during regeneration was detected using in situ hybridization. Fosl1 probe preparation and in situ hybridization were performed as previously reported in our group^62^. In brief, the whole open-reading frame of *X. tropicalis* fosl1 (accession number: XM_002939331.4) was amplified using cDNA templates from st42 tadpoles. Primers used for RT-PCR are as follows: *fosl1* Fw (BamH1): 5’-CGC GGA TCC ATG TAC AGA GAC TTC ACT GGA GCC TT-3’; *fosl1* Re (Xhol): 5’-CCG CTC GAG CTA AAG AGT CAG TAG GCT GTT AGA ACT T-3’. PCR fragments were then subcloned into pBlueScript II SK (+) plasmid and verified by sequencing. Plasmids were linearized and then used as templates for synthesis of digoxigenin-labeled antisense probes with T7 RNA polymerase (Roche, Indianapolis, IN). Whole-mount in situ hybridization was performed using digoxigenin-labeled antisense RNA probe and anti-digoxigenin monoclonal antibody labeled with alkaline phosphatase. Probe signals were developed using NBT/BCIP (Roche, USA).

### Statistical analysis

All statistics were calculated using the GraphPad Prism 8 Software. All data are presented as mean ± SEM. Among three or more groups, statistical analysis was performed using one-way analysis of variance followed by Dunnett’s multiple comparison tests. Comparisons between two groups were analyzed using unpaired Student’s *t* test. A *p* value of <0.05 was considered statistically significant.

### Reporting summary

Further information on research design is available in the Nature Research Reporting Summary linked to this article.

## Supplementary information

Supplementary Movie 1

Supplementary Movie 2

Supplementary Movie 3

Supplementary Movie 4

Supplementary Information

Reporting Summary

## Data Availability

The data that support the findings of this study are available from the corresponding author upon reasonable request. The gene expression data have been deposited in the NCBI Sequence Read Archive (BioProject accession: PRJNA401530).
