# Supplementary material for: Fosl1 is vital to heart regeneration upon apex resection in adult Xenopus tropicalis
Source: NPJ Regen Med. 2021 Jun 29;6:36. doi: 10.1038/s41536-021-00146-y (PMC8242016; doi:10.1038/s41536-021-00146-y)
Supplement: Supplementary file 5 — Supplementary Information [file 41536_2021_146_MOESM5_ESM.pdf]

Supplementary materials

Fosl1 is vital to heart regeneration upon apex resection in adult *Xenopus tropicalis*

Hai-Yan Wu, Yi-Min Zhou, Zhu-Qin Liao, Jia-Wen Zhong, You-Bin Liu, Hui Zhao, Chi-Qian Liang, Rui-Jin Huang, Kyu-Sang Park, Shan-Shan Feng, Li Zheng, Dong-Qing Cai, Xu-Feng Qi

Supplementary Figures

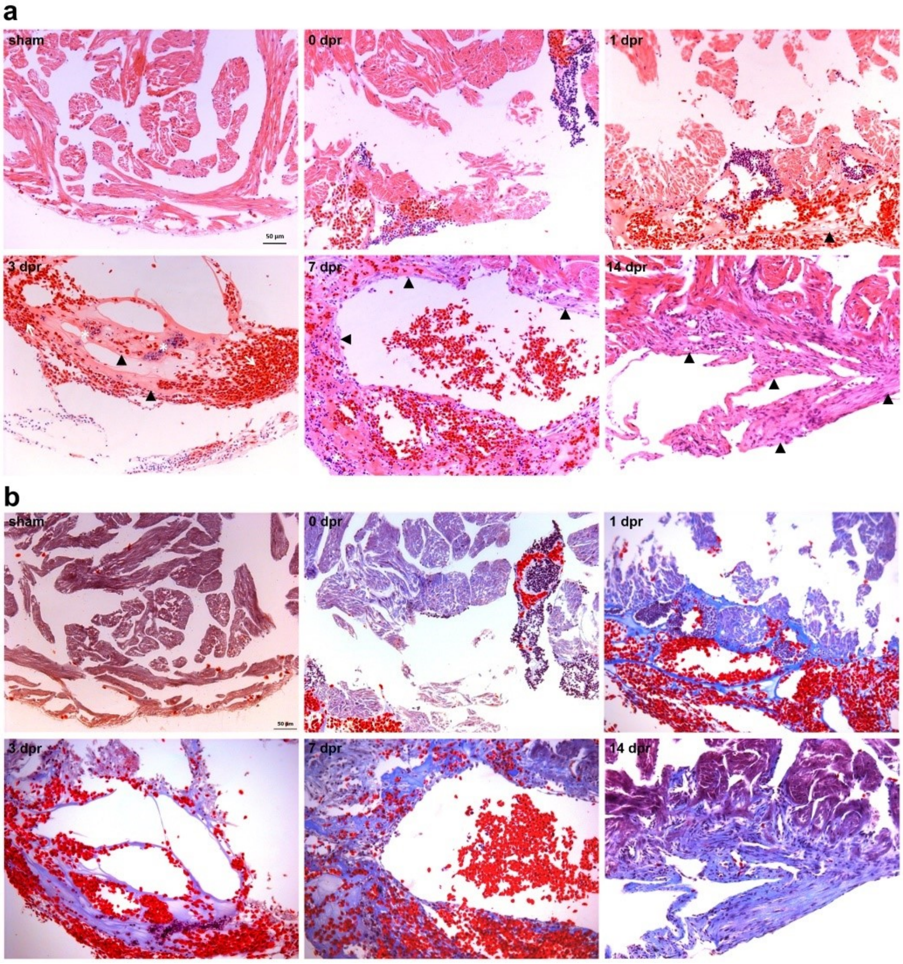

Supplementary Figure 1. Inflammatory response and fibrotic response during early stage of heart regeneration in *X. tropicalis*. a H&E-stained heart sections at higher magnification at 0 to 14 dpr. At 0 dpr, little monocytes (asterisks) and erythrocytes (arrow) were detected in the resection plane. At 1 to 3 dpr, resection plane was sealed by large blood clot consisting of robust monocytes (asterisks) and erythrocytes (arrow), accompanied by little fibrin (arrowhead). At 7 dpr, fibrin clot was surrounded by the infiltrating monocytes and erythrocytes. By 14 dpr, inflammatory response was gradually disappeared in the fibrin clot. **b** Masson trichrome-stained heart sections at higher magnification at 0 to 14 dpr. At 0 dpr, no fibrosis was detected around the wound. Fibrotic response was greatly increased in the resection plane at 1 to 14 dpr, and reached to the maximum level by 14 dpr, which was accompanied by the disappearance of inflammation (erythrocytes).

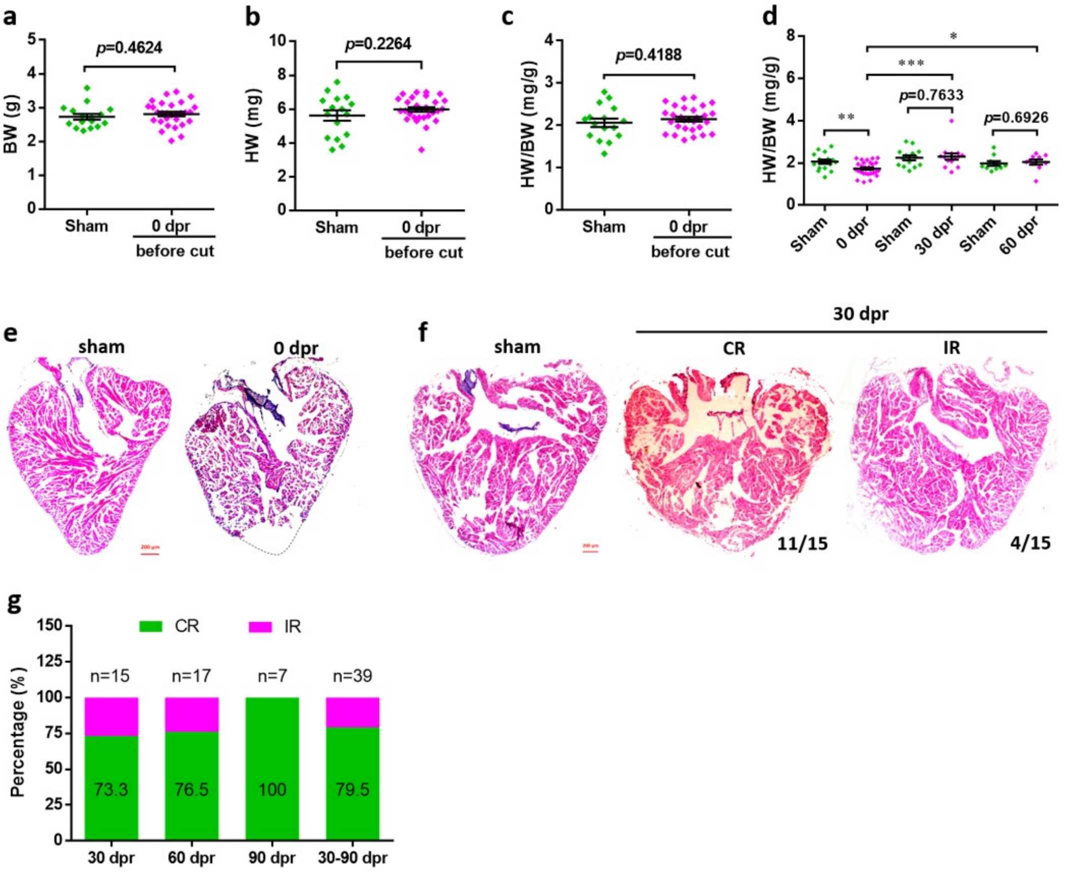

Supplementary Figure 2. Quantification of heart regeneration in *X. tropicalis*. a-c Quantification of body weight (BW), heart weight (HW) and HW/BW ratio of frogs in sham-operated and experimental groups before heart resection. Data are presented as mean ± SEM, n=16 for sham group, n=29 for experimental group (0 dpr, before resection). A Student's t test was used to determine statistical signification. **d** HW/BW ratio of frogs at 0-60 dpr compared with sham groups, showing powerful regeneration of the resected apex at 30 and 60 dpr. Data are presented as mean ± SEM (n=7-29 per group, similar with Figure 1d), *$p<0.05$, **$p<0.01$, ***$p<0.001$ versus sham (Student's t test). **e** Representative H&E-stained ventricle sections showing images of sham-operated heart (left) and resected heart (right). Dotted area denotes the resected ventricular apex. **f** Representative H&E-stained ventricle sections showing images of sham-operated heart and resected hearts at 30 dpr (CR, complete regeneration; IR, incomplete regeneration). **g** Quantification of CR and IR hearts in frogs at 30-90 dpr, showing about 73.3% CR hearts by 30 dpr.

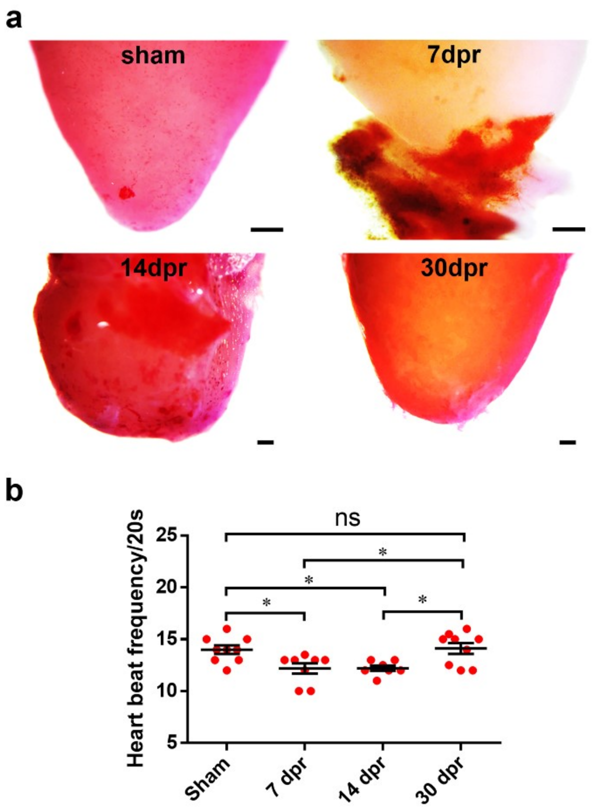

Supplementary Figure 3. Morphology and heart systolic function analysis of the regenerated heart in *X. tropicalis*. **a** Images of ventricle apex at 30 dpr showing the restoration of normal morphology compared with sham-operated hearts. **b** Quantification of heart rate at 30 dpr indicating the restoration of normal systolic function of regenerated heart. Relative heart rate is presented as percentages of sham-operated hearts. Data are presented as mean ± SEM (n=9 for sham, 8 for 7dpr, 7 for 14dpr, 9 for 30dpr), *p<0.05. A Student's t test was used to determine statistical signification.

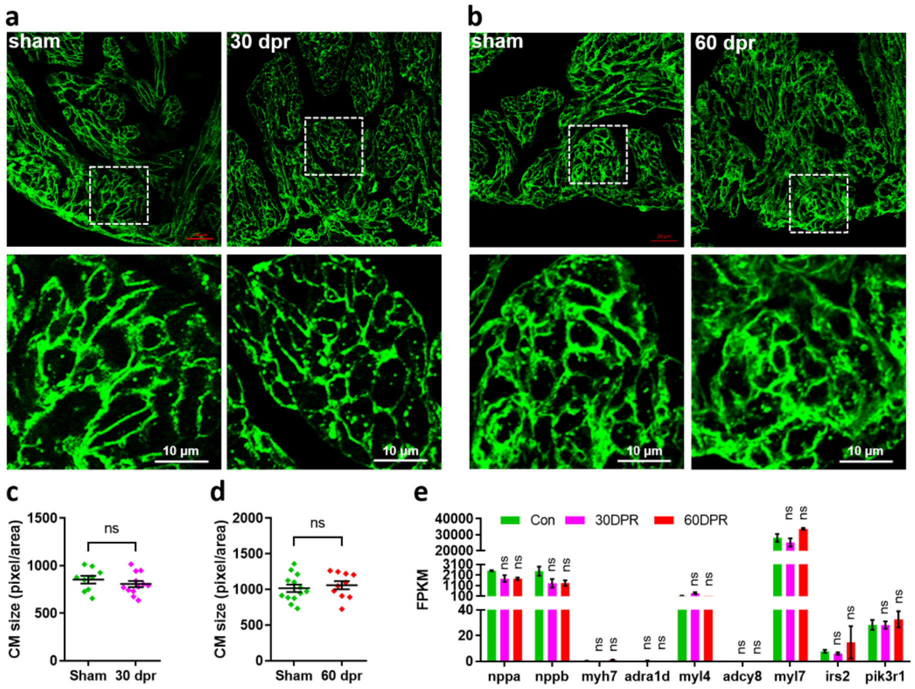

Supplementary Figure 4. Heart regeneration in *X. tropicalis* doesn't result from cardiomyocyte hypertrophy. **a**, **b** Representative wheat germ agglutinin (WGA) staining images of apical myocardium in sham and resected hearts at 30 dpr (a) and 60 dpr (b). Lower panels are the magnified images. **c**, **d** Quantification of cardiomyocyte size in apex of sham and resected hearts at 30 dpr (c, *n*=9 for sham, and 12 for 30 dpr) and 60 dpr (d, *n*=13 for sham, and 10 for 60 dpr). Data are presented as mean ± SEM (Student's *t* test). **e** Ventricular apexes were analyzed for hypertrophy-related genes expression by using FPKM levels from RNA-seq, showing no significant differences at 30-60 dpr compared with sham. Data are presented as mean ± SEM (*n*=3 per group). A One-way ANOVA followed by Dunnett's test was used to determine statistical signification. Ns means no significant difference.

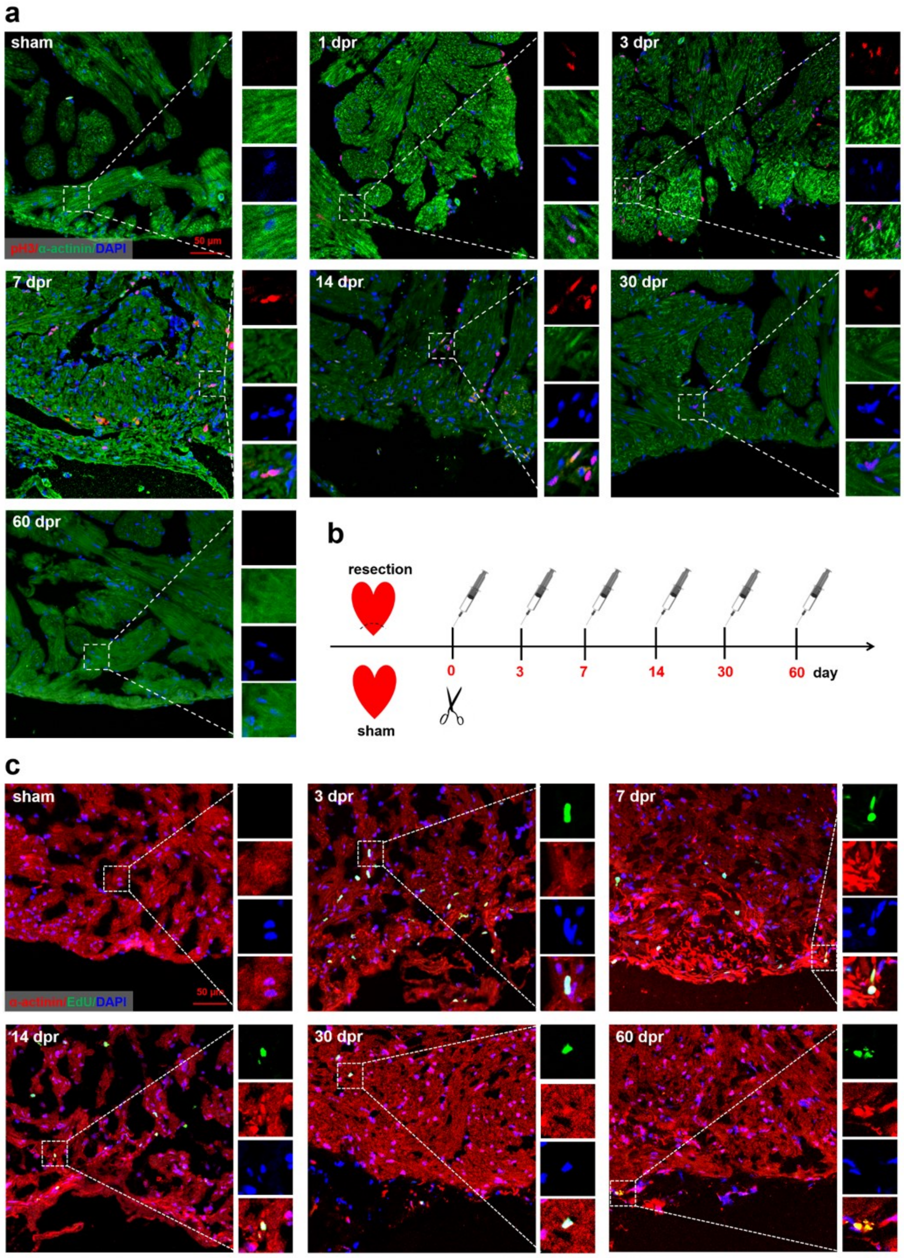

Supplementary Figure 5. Cardiomyocyte proliferation accompanies heart regeneration in *X. tropicalis*. a Representative confocal images showing increased CMs mitoses in ventricle apex at 1 to 14 dpr, which was followed by the greatly decrease in CMs mitoses at 30 to 60 dpr. Right panels at high magnification indicate the representative pH3-positive CMs boxed by dotted line in left panels. Red indicates pH3; green, α-actinin; blue, nuclei. **b** Schematic of EdU injection to label proliferating CMs. **c** EdU-positive CMs in the ventricle apex following EdU injection indicating the nuclear incorporation of EdU (green) in proliferating CMs. Red indicates α-actinin; blue, nuclei. Right panels at high magnification indicate the representative EdU-positive CMs boxed by dotted line in left panels.

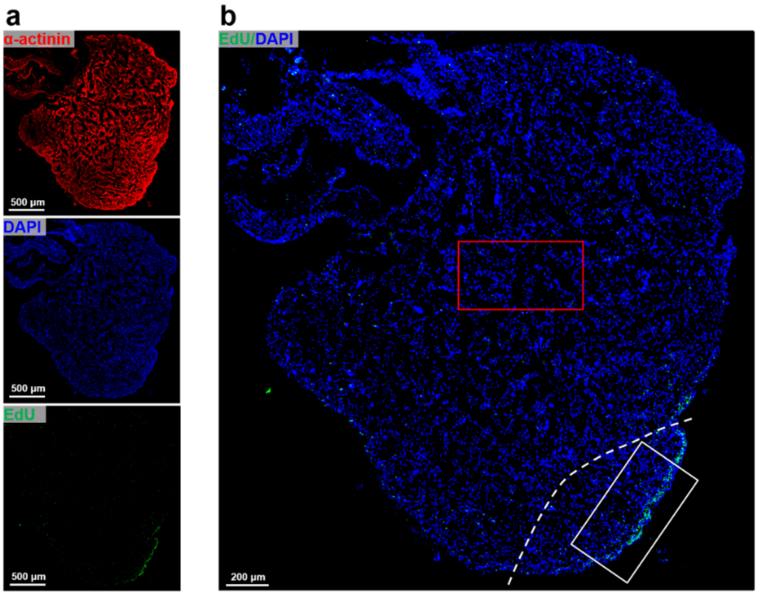

Supplementary Figure 6. Representative whole images of the regenerating *X. tropicalis* heart at 30 dpr (related to Fig. 3). a Representative image of whole heart at 30 dpr in low-magnification. Upper panel, α-actinin staining in red; Middle panel, DAPI staining in blue; Lower panel, EdU staining in green. **b** Representative image of whole heart at 30 dpr in high-magnification with DAPI (blue) and EdU (green) double staining. White dotted line denotes the amputation plane. White rectangle denotes the regenerated ventricle apex with maximum proliferating cardiomyocytes. Red rectangle denotes the remote zone located in the middle of ventricle.

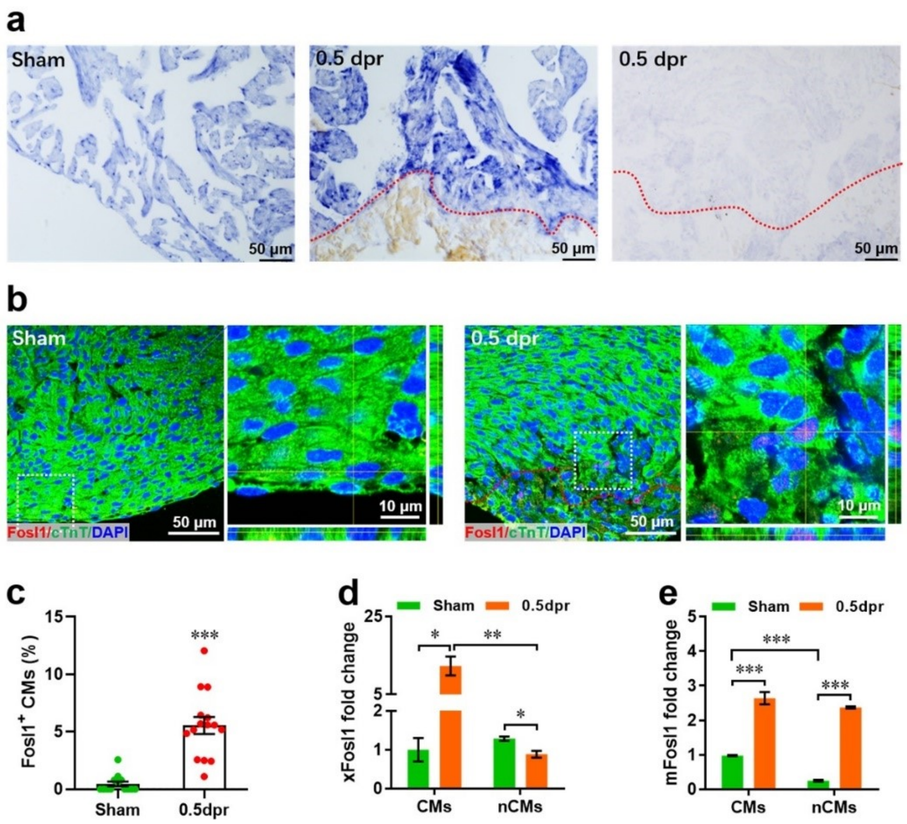

Supplementary Figure 7. Expression patterns of Fosl1 during heart regeneration. a xFosl1 expression analyzed by *in situ* hybridization in sham or injured *X. tropicalis* heart at 0.5 dpr (right panel, sense probe of xFosl1). **b**, **c** Representative image (b) and quantification (c) of Fosl1-positive cardiomyocytes in sham-operated and injured neonatal mouse hearts at 0.5 dpr (*n*=15 sections from 5 hearts per group). **d** qPCR validation of xFosl1 expression in cardiomyocytes (CMs) and non-cardiomyocytes (nCMs) isolated from sham-operated and injured *X. tropicalis* hearts at 0.5 dpr (*n*=3 per group). **e** qPCR validation of mFosl1 expression in cardiomyocytes (CMs) and non-cardiomyocytes (nCMs) isolated from sham-operated and injured neonatal mouse hearts at 0.5 dpr (*n*=3 per group). Data are presented as mean ± SEM (*n*=3 per group), **p*<0.05, ***p*<0.01, ****p*<0.001 (Student's *t* test).

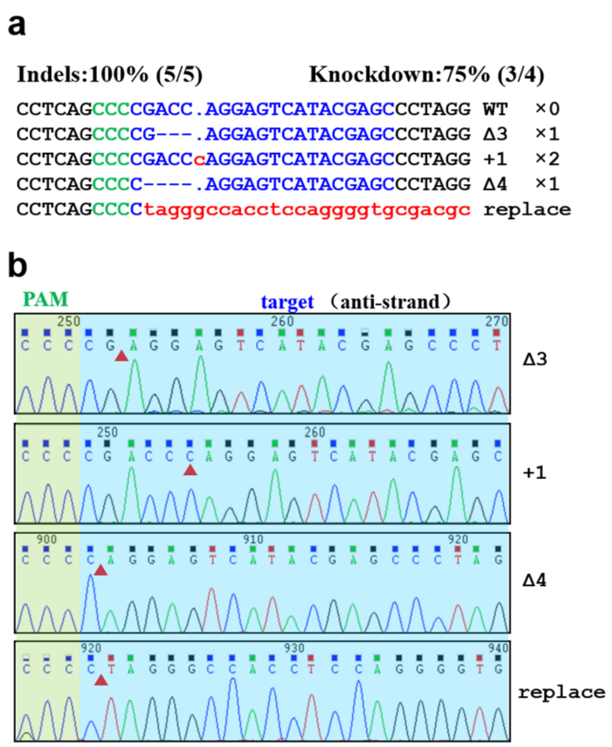

Supplementary Figure 8. Evaluation of *Fosl1* knockdown in H9c2 cells transfected with LentiCRISPRv2. H9c2 cells were infected with LentiCRISPRv2 viruses and selected by puromycin to establish single cell colonies by serial dilution. **a** DNA sequencing data of five single cell colonies revealed highly efficient indel generation (100%) and frameshift mutation (75%). Wild-type (WT) sequence is shown at top with target site in blue and the PAM sequence in green. **b** Representative chromatograms of the DNA sequencing assay from stable cell line confirmed indel generation (red arrowhead) and frameshift mutation (3 out of 4 indels).

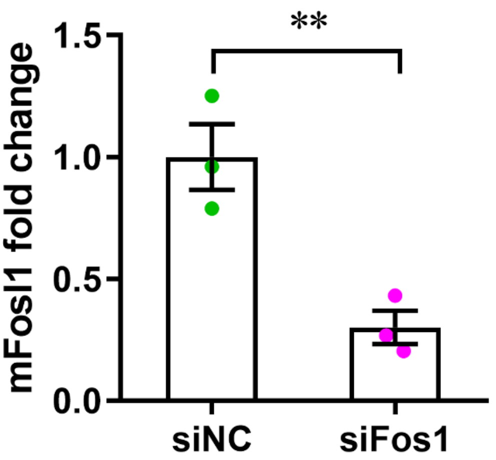

Supplementary Figure 9. qPCR validation of mouse Fosl1 (mFosl1) silencing in primary cardiomyocytes isolated from neonatal mice (*n*=3 each). Data are presented as mean ± SEM (*n*=3 each), ***p*<0.01 versus control (Student's *t*-test),

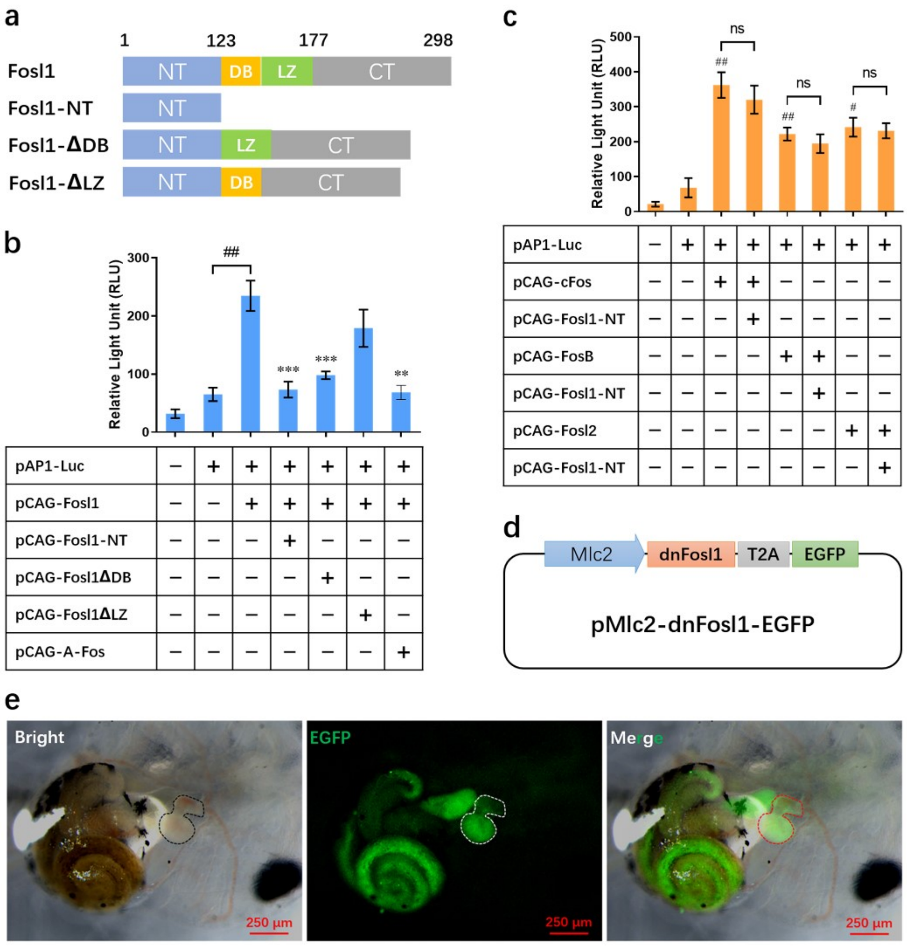

Supplementary Figure 10. Construction of dominant-negative Fosl1 (dnFosl1) and transgenic *X. tropicalis* line. a Schematic representation of the deletion mutants of *X. tropicalis* Fosl1 (XP_002939377). The numbers on the top of the scheme indicate the amino acid position. Fosl1-NT, N-terminus (aa 1-123). Fosl1ΔDB, mutant removing the DNA-binding (DB) domain (aa 124-147). Fosl1ΔLZ, mutant removing the leucine zipper (LZ) domain (aa 149-177). **b** 293T cells were transfected with pAP1-GLuc and pcDNA-Fosl1 with or without mutants for 48 h, followed by luciferase activity assay. Data are presented as mean ± SEM (n=5 each), **p<0.01, ***p<0.001 versus Fosl1-derived luciferase activity, $^{\#\#}p$<0.01. (Student's t-test). **c** 293T cells were transfected with pAP1-GLuc in together with cFos, FosB, or Fosl2 plasmids with or without pcDNA-Fosl1-NT for 48 h, followed by luciferase activity assay. Data are presented as mean ± SEM (n=5 each), $^{\#}p$<0.05, $^{\#\#}p$<0.01 versus pAP1-GLuc alone (Student's t-test). Ns, no significant difference. **d** Schematic illustrating the transgenic plasmid used for construction of *Tg(Mlc2-dnFosl1-T2A-EGFP)* line. **e** Representative images of *Tg(Mlc2-dnFosl1-T2A-EGFP)* line in tadpole stage. Dotted line denotes the heart with EGFP reporter gene expression.

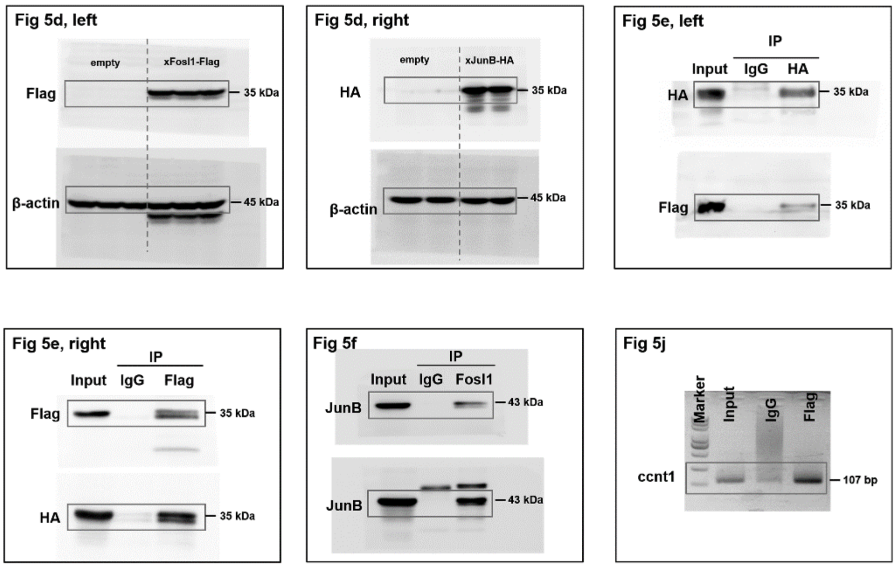

Supplementary figure 11. Uncropped blots relate to Figure 5.

Supplementary Table 1. Sequences of siRNAs targeting rat *Fosl1* gene used in this study.

Target gene	NCBI Reference No.	siRNAs	
		Name	Sequence (5’-3’)
NC	/	siNC	Purchase from BiboBio
rFosl1 (H9c2)	NM_012953.1	siFosl1	ACCCGTACTTGAACCGGAA
mFosl1 (mouse primary CMs)	NM_010235.2	siFosl1	GCAGCGAGAGATTGAAGAGCT

Supplementary Table 2. Primer sequences for real-time PCR analysis in *X. tropicalis*.

Gene	NCBI Reference No.	Primer sequence (5'-3')	
		Forward primer	Reverse primer
rcc2	XM_002942127.3	AGCAACAAGCTGCCTATCGT	TGTCATTACGCCCCCAACTC
fosl1	XM_002939331.4	CAAGTGTACGCCCCCTGAAT	GTTTGTCTGTCTCTGCCTGC
rgcc	NM_001102798.1	GTGCGAATTTGATGCGGTCA	CCAGTTTTGCTTTGTGAGGGG
sik1	XM_018090369.1	TTTCACAGGCCAGAGAGCTG	ATGGCGCGGATCTTACTGAG
fos	NM_001016200.2	GCTTACAGCCGATCTAGCGT	AGGTCATCAGTCTCCGCTTG
anapc11	NM_001011188.1	CATATTTGCAGGAGGTGCTCG	CCTGACGACACATAGGGCAG
jun	XM_018093560.1	TATACTGGGCACACGCACAC	AGCACCAGCTTTGTGTCTCT
junb	NM_001044490.1	CAGAATTGTTGCAGCGCAGT	CTCTGTGTGTGCGCTGTTTC
jund	XM_002937933.4	TCTCAGGCTCCCCTCTACAC	GCTTTCACAAAGCCCTCTGC
myc	XM_012964474.2	TCACCTTGCCAAGACCTCATGTTG	TTCCTCATCACAGTCTTCATCCTCG
cdk2	NM_001008135.1	TCATGAGGTTGTCACCCTGT	AGTCCTGTCGGACCCACTTA
cdk4	NM_001016742.1	TCTCTACGGTCAGGGAGGTC	CTCGGTGCACGATGCAATTC
cdk8	NM_203915.1	TCGAGCCCCAGAGTTATTGC	TTGGTGTACGTATTCCTGCGG
cdk9	XM_012968193.2	ACATCGACAGACGGGGAAGA	AGCCTGCTAGATCATGCTCAC
cdk12	XM_012971492.1	AAGTCGTGGATGCACCTGG	CTCCGGAGGCAGGATACGAG
cdk13	XM_002940799.4	TGCACGGCTCTACAGTTCAG	CTCACCCAGGATACAGCCAC
cdk17	XM_018091901.1	GAGCTGGCAGAACAAATGACC	TGGAGGAAGGAGTGCATACTG
ccnc	XM_012962841.2	TGATTGCTCTTGCCTGTCTG	CCCAGAGCTTTGGCTTCCAT
ccne2	NM_001016267.2	TGTCAAGAAGAAGTGGCCGT	CCTGTATCGTGAGAACTGCGA
ccnk	NM_001078855.1	ATGGTGGGAGCAGTTTGTCC	GTGAAGTAGGCAAAGGTCGC
ccnl1	XM_012962551.2	CCGTCTATGTCAGATGGCCT	GGGACTGGGGTCCTTTTTGT
ccnl2	NM_001030452.1	GGGCTTTTGTGTGCATGTGA	GCAGAGGGATCTCTAGCGT
ccnt1	XM_018091104.1	CTGGACTGGACGCCGATAAG	GGTCCCACAGAGTTACGGTG
ccnt2	XM_004917646.3	TGGCCCATGCCTGTCTTAAC	TAGTGAGGTGCAGGCTGTTG
ccny	XM_002935160.4	TGTGGAACGTGGACTACTGC	ACAAGCGAGAAATGGCCTCA
chek1	XM_012966987.2	ATGCTTCTGAGCAGCCAGTT	ACTCCAGCCCATCACCCTTA
chek2	NM_001126524.1	TCTCAACAATCTCAGGGCACC	TGTACCTCTCCGTCTGGTTT
cdkn1a	XM_002935778.4	GGGACCACATCACATGAGCG	TGCTTCCTCGTTGGATCTCTG
cdkn2d	NM_001079371.1	TGCCAGGGGAGACCTATTAGA	GCTCCATGCTGGACCAAAAC
gapdh	XM_012966386.2	TATCTGCTCACGCCACGATT	ATCAACCCCTCTCGTTTCTG

Supplementary Table 3. Primer sequences for real-time PCR analysis in H9c2 cells.

Gene	NCBI Reference No.	Primer sequence (5'-3')	
		Forward primer	Reverse primer
Fosl1	NM_012953.1	ATGTACCGAGACTTCGGGGA	GCTCGTATGACTCCTGGTCG
Gapdh	NM_017008.4	TGTGTCCGTCGTGGATCTGA	CCTGCTTCACCACCTTCTTGA

Supplementary Table 4. Primer sequences for real-time PCR analysis in mouse.

Gene	NCBI Reference No.	Primer sequence (5'-3')	
		Forward primer	Reverse primer
Fosl1	NM_010235.2	CGGCCAGGAGTCATACGAG	ATTTTGCAGATGGGGCGATG
JunB	NM_008416.3	AGGCAGCTACTTTTCGGGTC	GCGTCACGTGGTTCATCTTG
Gapdh	NM_001289726.1	TGTGTCCGTCGTGGATCTGA	CCTGCTTCACCACCTTCTTGA

Supplementary Table 5. Predicted FOSL1::JUNB binding sites in the promoter regions of *X. tropicalis* target genes.

Matrix ID	Name	Target genes	Score	Relative score	Start	End	Strand	Predicted sequence
MA1137.1	FOSL1::JUNB	*cdk4*	9.25956	0.859216202012	-50	-38	-	aagtgacgcacac
MA1137.1	FOSL1::JUNB	*cdk9*	7.07591	0.808453743341	-1244	-1232	+	cactgacttatgg
MA1137.1	FOSL1::JUNB	*cdk12*	7.16858	0.810607975818	-1347	-1335	+	aaatgatgcagaa
MA1137.1	FOSL1::JUNB	*chek2*	10.2399	0.882006216903	-929	-917	+	ttctgactaatta
MA1137.1	FOSL1::JUNB	*ccnc*	9.37966	0.862008035868	-396	-384	-	ctatgacacacag
MA1137.1	FOSL1::JUNB	*ccnl2*	7.01876	0.807125189132	-1412	-1400	-	agaggactcagtg
MA1137.1	FOSL1::JUNB	*ccnt1*	8.77859	0.848035165697	-1398	-1386	+	ttatgactattct

The promoters (-2,000 to -1, upstream of TSS) of target gene were predicted by JASPAR 2018 online software. The binding site with highest score was listed and further analyzed by luciferase report gene system.

Legends for supplementary videos

Supplementary video 1. Heart beating of *X. tropicalis* in sham-operated group. Hearts were extracted from *Xenopus tropicalis* frogs (6 months old) in sham-operate group, and heart beating frequency within 20 seconds were captured by Leica M205FA stereo fluorescence microscope. Scale bar, 1 mm.

Supplementary video 2. Heart beating of *X. tropicalis* in resected group at 7 dpr. Hearts were extracted from *Xenopus tropicalis* frogs (6 months old) in resected group at 7 dpr, and heart beating frequency within 20 seconds were captured by Leica M205FA stereo fluorescence microscope. Scale bar, 2 mm.

Supplementary video 3. Heart beating of *X. tropicalis* in resected group at 14 dpr. Hearts were extracted from *Xenopus tropicalis* frogs (6 months old) in resected group at 14 dpr, and heart beating frequency within 20 seconds were captured by Leica M205FA stereo fluorescence microscope. Scale bar, 1 mm.

Supplementary video 4. Heart beating of *X. tropicalis* in resected group at 30 dpr. Hearts were extracted from *Xenopus tropicalis* frogs (6 months old) in resected group at 30 dpr, and heart beating frequency within 20 seconds were captured by Leica M205FA stereo fluorescence microscope. Scale bar, 1 mm.
